# Extensive parasite transmission and variation in a functional receptor associated with drug resistance in endemic *Schistosoma mansoni*

**DOI:** 10.1126/sciadv.adt3721

**Published:** 2026-07-17

**Authors:** Duncan J. Berger, Sang-Kyu Park, Thomas Crellen, Tushabe John Vianney, Narcis B. Kabatereine, James A. Cotton, Richard Sanya, Alison Elliott, Edridah M. Tukahebwa, Moses Adriko, Claire J. Standley, Anouk Gouvras, Safari Kinung’hi, Muriel Rabone, Aidan Emery, Poppy H. L. Lamberton, Bonnie L. Webster, Fiona Allan, Sarah Buddenborg, Matthew Berriman, Jonathan S. Marchant, Stephen R. Doyle, Joanne P. Webster

**Affiliations:** ^1^ Wellcome Sanger Institute, Wellcome Genome Campus, Hinxton, Cambridgeshire, UK.; ^2^ Department of Cell Biology, Neurobiology and Anatomy, Medical College of Wisconsin, Milwaukee, WI, USA.; ^3^ Department of Infectious Disease Epidemiology, School of Public Health, Imperial College Faculty of Medicine, London, UK.; ^4^ Division of Biomedical Engineering, University of Glasgow, Glasgow, UK.; ^5^ Vector Borne & Neglected Tropical Disease Control Division, Ministry of Health, Kampala, Uganda.; ^6^ School of Biodiversity, One Health, and Veterinary Medicine, University of Glasgow, Glasgow, UK.; ^7^ Immunomodulation and Vaccines Programme, Medical Research Council/Uganda Virus Research Institute.; ^8^ Center for Global Health Science and Security, Georgetown University, 3900 Reservoir Rd NW, Washington, DC, USA.; ^9^ Global Schistosomiasis Alliance, Podium Space–Ealing Cross, 85 Uxbridge Road, London, UK.; ^10^ National Institute for Medical Research (NIMR) Mwanza Centre, P.O. Box 1462, Mwanza, United Republic of Tanzania.; ^11^ Wolfson Wellcome Biomedical Laboratories, Department of Life Sciences, The Natural History Museum, London, UK.; ^12^ Global Centre for Neglected Tropical Disease Research (GCNTDR), Herts, UK.; ^13^ University of St Andrews, Pelagic Ecology Research Group, Scottish Oceans Institute, St. Andrews, UK.; ^14^ Department of Pathobiology and Population Sciences, Royal Veterinary College, University of London, Herts, UK.

## Abstract

Mass drug administration (MDA) with praziquantel is the cornerstone of schistosomiasis control and elimination efforts. To assess how long-term MDA affects parasite populations, we analyzed whole-genome sequence data from 570 *Schistosoma mansoni* samples and the closely related *Schistosoma rodhaini* across eight countries, combining new parasite material with publicly available sequence data. We observed a broad-scale genetic structure between countries alongside evidence of extensive long-distance transmission. Functional profiling of the recently identified transient receptor potential melastatin ion channel, *Sm.*TRPM_PZQ_, revealed four naturally occurring variants associated with reduced praziquantel sensitivity, indicating standing variation for resistance. Analyses of parasite infrapopulations collected from people pre– and post–praziquantel treatment further identified instances of treatment failure, supporting the potential for praziquantel resistance. As schistosomiasis is targeted for elimination as a public health problem, with interruption of transmission in selected regions by 2030, our study provides a comprehensive genomic framework for endemic populations, highlights an approach to detect potential resistance, and endorses the development of precision surveillance and adaptive treatment strategies.

## Teaser

Population genomics and functional genetics reveal variants associated with praziquantel response in *Schistosoma mansoni*.

## INTRODUCTION

Schistosomiasis is a major neglected tropical disease that currently infects more than 250 million people across 78 endemic nations (*1*, *2*). Infections are prevalent among children, including preschool-aged children, as well as adults in low- and middle-income countries, with 90% of infections occurring in sub-Saharan Africa. The etiological agents of schistosomiasis are freshwater snail-borne parasitic trematodes of the genus *Schistosoma* (principally *Schistosoma mansoni, Schistosoma japonicum*, and *Schistosoma haematobium* for human schistosomiasis). These parasitic worms reside in the host’s blood vessels, often for years, where they lay eggs; many of these eggs become trapped in host tissues, resulting in a spectrum of pathologies, including anemia, stunted growth, genital lesions, fever, irreversible organ damage, and death (*3*). Recognizing the severe and widespread impact of this disease, in 2002, the World Health Organization (WHO) endorsed praziquantel monotherapy, the sole drug available, distributed as part of mass drug administration (MDA) programs, as the primary strategy for schistosomiasis control (*4*, *5*). In 2020 alone, 76.9 million people (representing 44.9% of those requiring treatment), predominantly school-aged children, were treated with praziquantel for schistosomiasis (*6*). Such treatment campaigns resulted in an overall decrease in the prevalence of schistosomiasis among school-aged children by ∼60% (*7*, *8*). On the basis of such successes, the WHO subsequently launched its 2021 to 2030 neglected tropical disease roadmap and revised *WHO Guideline for the Control and Elimination of Schistosomiasis* (*1*). These set ambitious goals to eliminate schistosomiasis as a public health problem in all endemic countries (defined as reducing the proportion of heavy-intensity infections to <1%) and complete interruption of transmission in selected regions by 2030, primarily through the escalating use of a single oral dose of praziquantel (40 mg/kg) via MDA (*9*).

Nevertheless, despite substantial progress toward the WHO goals in general (*7*, *10*, *11*), prevalence reductions have proven reversible over short timescales across several regions, and despite years of repeated high-coverage MDA, persistent hotspots of infection remain (*12*–*15*). Ongoing surveillance projects have also revealed substantial heterogeneity in infection prevalence, intensity, and morbidity within and between endemic regions (*16*–*18*). This includes potential variability in the parasite’s response to praziquantel, which, given the current lack of vaccines or alternative antischistosomal drugs, represents a major threat to the control and elimination of schistosomiasis (*19*–*21*). Such MDA programs are expected to markedly alter patterns of schistosome transmission and exert strong selective pressures for the evolution of praziquantel resistance. The emergence of resistance has already rendered the previous drug, oxamniquine, ineffective for treating human *S. mansoni* infections across Africa (*22*). Likewise, heritable resistance has already emerged or established, particularly across the veterinary helminths, to every other class of anthelmintic used (*23*–*26*). There is clear evidence that resistance to praziquantel can be rapidly selected for in laboratory *S. mansoni* settings (*27*–*30*), and resistance to praziquantel has recently emerged under field settings for other parasites, such as the tapeworms *Dipylidium caninum* in dogs and cats (*31*, *32*) and potentially also *Anoplocephala perfoliata* in horses (*33*). However, despite more than 20 years of human MDA, there is limited evidence of established praziquantel resistance in endemic *S. mansoni* populations (*30*). Sustained praziquantel administration appears to have had limited to no impact on the parasite population genetic structure or diversity (*34*–*37*), although reduced praziquantel efficacy has been documented for Ugandan *S. mansoni* populations among children under the longest MDA histories (*20*) and, more recently, in Senegalese *Schistosoma curassoni* and *Schistosoma bovis* populations of livestock exposed to frequent praziquantel use and misuse (*38*). Furthermore, until recently, we neither knew the precise mode of action of praziquantel against schistosomes nor had any molecular markers to monitor potential praziquantel resistance among natural *Schistosoma* spp. populations. Recent efforts have, however, identified the molecular target of praziquantel, a transient receptor potential (TRP) melastatin ion channel (*Sm*.TRPM_PZQ_) (*39*–*42*). This finding therefore permits evaluation of praziquantel efficacy and resistance risk, although such resistance-conferring alleles have only been identified to date from a single isolate from endemic populations (*41*). Characterizing the transmission and recent evolution of schistosome populations is, therefore, of broad epidemiological importance as a means to understand how parasite populations are structured and how they are changing in response to interventions aimed at controlling schistosomiasis.

Here, we have used whole-genome sequencing to characterize genomic variation across globally dispersed populations of the major human-infecting species *S. mansoni* (and the closely related outgroup species, *Schistosoma rodhaini*) (*43*–*45*). Our analyses focus on populations within endemic regions of East Africa, primarily those found around Lake Victoria, one of the largest foci of schistosomiasis infections and a target of long-term MDA efforts (*7*, *20*). We aimed to quantify variation within the previously unidentified candidate praziquantel resistance locus, *Sm*.TRPM_PZQ_ (*40*, *41*, *46*), to determine the prevalence of potential resistance-conferring mutations. This also included extensive whole-genome sequencing of *S. mansoni* from within individual hosts before and after praziquantel administration to quantify the immediate genetic impact of treatment. These data raise important implications regarding the standing variation of potential praziquantel resistance in human schistosome populations and represent an important resource for assessing the efficacy of current interventions and guiding future treatment strategies.

## RESULTS

### The genetic diversity of geographically dispersed isolates supports high transmission in endemic regions

**Fig. 1.**
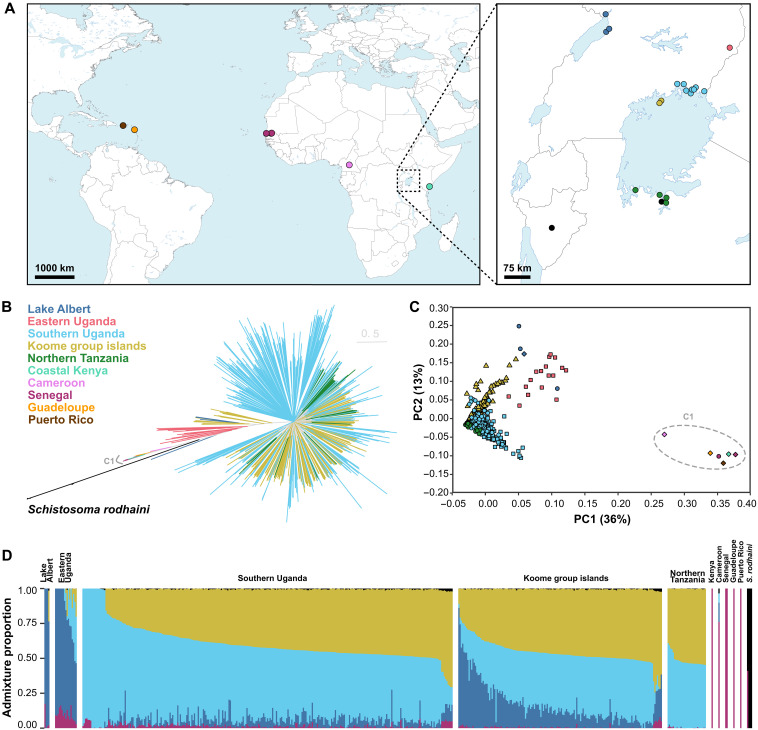
Population structure of *Schistosoma* accessions. (**A**) Global distribution of the 574 *Schistosoma* accessions used in this study. Sampling locations are colored by geographical divisions: Puerto Rico (PR; brown), Guadeloupe (GP; orange), Senegal (SN; dark purple), Cameroon (pink), Coastal Kenya (KE; teal), Lake Albert (dark blue), Eastern Uganda (red), Southern Uganda (light blue), Koome group islands (yellow), and Northern Tanzania (dark green). Black points represent the sampling coordinates of *S. rodhaini* accessions. (**B**) The maximum-likelihood phylogenetic tree was inferred using 188,923 autosomal SNPs and all 574 accessions. Branches are colored according to the subdivisions described in (A), and the tree is rooted at *S. rodhaini*. The highlighted clade “C1” represents all West African (Senegalese and Cameroonian), Caribbean (Puerto Rican and Guadeloupean), and Coastal Kenyan accessions. (**C**) Principal components (PC) analysis of genetic differentiation between 505 unrelated *S. mansoni* accessions using 214,445 autosomal SNPs. Points are colored and shaped on the basis of the groups described in (A). (**D**) ADMIXTURE plots illustrating the inferred ancestry of 505 unrelated *S. mansoni* and four *S. rodhaini* accessions. Here, we assume that five populations (K) are present, inferred using 10-fold cross-validation and standard error estimation on the basis of 250 bootstraps. *y*-axis values show the admixture proportions for each accession, and colors for each population were assigned on the basis of the majority ancestry of each geographical division.

We analyzed whole-genome sequence data from 574 *Schistosoma* samples (*n* = 570 *S. mansoni* and *n* = 4 *S. rodhaini*; hereafter all referred to as accessions) isolated from eight countries, including 207 new *Schistosoma* samples sequenced for this study (Fig. 1A, Table 1, and table S1) (*43*–*45*). Most accessions were derived from Lake Victoria (88.3%), a major focus of *S. mansoni* infection. These included published sequence data from miracidial samples isolated from infected children between 2014 and 2017 from either the Koome and Damba Islands (hereafter referred to as the Koome Islands; *n =* 174) (*45*) or Southern Uganda (*n =* 164) (*43*), with a median of two isolates sequenced per child (range, 1 to 11). We sequenced an additional 164 miracidia from just three children from Southern Uganda (*n =* 89, 51, and 23 miracidia per child), including both pre–praziquantel treatment (*n* = 82) and post–praziquantel treatment (*n* = 82) sampling (*20*). The only other Lake Victoria accessions were derived from cercariae shed from snails captured in Northern Tanzania (*n =* 31) or Southern Uganda (*n* = 2). Of the remaining samples, two were cercariae from the shoreline of Lake Albert, four were cercariae from a passaged strain originally isolated in Lake Albert, 17 were miracidia collected from children living in Eastern Uganda, and 10 were published adult-stage isolates of laboratory-passaged strains originally sampled from Kenya (*n =* 1), Senegal (*n =* 3), Cameroon (*n* = 1), Guadeloupe (*n =* 4), or Puerto Rico (*n =* 1) (*44*). As an outgroup, we included four *S. rodhaini* accessions, two cercarial isolates from Tanzania, and two adult-stage isolates of passaged laboratory strains originally sampled in Burundi. We mapped sequence reads from all 574 accessions to the *S. mansoni* reference genome, and after variant calling and quality control, we identified 35,146,249 single-nucleotide polymorphisms (SNPs) and 6,632,156 indels across all accessions (table S2).

**Table 1.** Sample information and history of praziquantel treatment. This study included 574 accessions, sequenced from parasite samples derived from snail hosts (cercariae), humans (miracidia), or laboratory-passaged strains (adults). Publicly available data (*n* = 339) and new sequencing data (*n* = 208; defined as “herein”) were generated for this study. Miracidial accessions were sampled pretreatment with praziquantel (40 mg/kg) or 4 weeks posttreatment.

Species	Country	Region	Development stage			Sampling stage			Reference
			Adults	Cercariae	Miracidia	Pretreatment	Posttreatment	Not applicable	
*Schistosoma mansoni*	Uganda	Lake Albert	1	6	0	0	0	7	Herein; (*20*)
	Uganda	Eastern Uganda	0	0	17	17	0	0	(*43*)
	Uganda	Southern Uganda	1	2	328	201	127	3	Herein; (*43*)
	Uganda	Koome Islands	0	0	174	123	51	0	(*45*)
	Tanzania	Northern Tanzania	0	31	0	0	0	31	Herein
	Kenya	Coastal Kenya	1	0	0	0	0	1	(*20*)
	Cameroon		1	0	0	0	0	1	(*20*)
	Senegal		3	0	0	0	0	3	Herein; (*20*)
	Puerto Rico		1	0	0	0	0	1	(*20*)
	Guadeloupe		4	0	0	0	0	4	(*20*)
*Schistosoma rodhaini*	Tanzania	Northern Tanzania	0	2	0	0	0	2	Herein
	Burundi		2	0	0	0	0	2	Herein; (*20*)

To characterize the population structure, we constructed a maximum-likelihood phylogeny and performed principal components analysis on unlinked autosomal variants. These analyses distinguished accessions sampled in East Africa from those in West Africa and the Caribbean (Fig. 1, B and C). The only exception was a single laboratory-passaged isolate from coastal Kenya that clustered with West African accessions, consistent with previous analyses of this sample (*44*, *47*). Within Eastern Africa, populations from Lake Victoria, Lake Albert, and Eastern Uganda formed distinct but partially overlapping clusters. Pairwise measures of the fixation index (*F*_ST_) showed limited population differentiation between Lake Victoria, Lake Albert, and Eastern Uganda (range *F*_ST_ = 2.49 × 10^−2^ to 4.59 × 10^−2^) and negligible differentiation between Lake Victoria populations despite the geographic separation of up to 300 km (range *F*_ST_ = 2.41 × 10^−3^ to 4.07 × 10^−3^; fig. S1A). East African populations had comparable levels of nucleotide diversity (median π = 2.35 × 10^−3^ to 3.35 × 10^−3^; fig. S1B) and effective population size (*N*_e_) estimates (*N*_e_ = 58,004 to 64,063; fig. S2 and table S3), suggesting shared recent population histories. Analyses of population ancestry for individual accessions identified similar population compositions across all Lake Victoria populations with variable ancestry contributions from Lake Albert and West African populations (Fig. 1D and table S4). Accessions from Eastern Uganda displayed mixed ancestry, with contributions from Lake Victoria, Lake Albert, and West African populations, suggesting migration from multiple regions.

Ancestry analyses revealed low levels of *S. rodhaini* admixture (range: 0.00 to 2.32%) across most unrelated *S. mansoni* accessions (Fig. 1D and table S4), potentially indicative of interspecific hybridization. In East Africa, *S. mansoni* and *S. rodhaini* are broadly sympatric and share both intermediate and definitive hosts, with *S. mansoni* infecting both specific primate and rodent species and *S. rodhaini* infecting rodents, allowing opportunities for interspecies pairings and hybridization (*48*, *49*). Incomplete lineage sorting, where ancestral genetic variation persists in descendant lineages after speciation, could provide an alternative explanation for the low levels of *S. rodhaini* admixture, given that the two species are very closely related. However, because of the small number of *S. rodhaini* isolates included in this study (*n* = 3) and the lack of a nonadmixed, outgroup population, the incidence of recent admixture and/or historic introgression was not investigated further.

### Functionally relevant genomic variation influences praziquantel sensitivity

A TRP melastatin ion channel (*Sm.*TRPM_PZQ_; *Smp_246790*) has recently been proposed as the target of praziquantel (*39*–*42*). This candidate gene underlies variation in praziquantel susceptibility, at least within schistosome laboratory populations (*39*–*41*, *50*). Mutagenesis of key residues within the praziquantel binding site of *Sm.*TRPM_PZQ_ [located in the transmembrane voltage sensor–like domain (VSLD)] resulted in the loss of sensitivity to praziquantel (*40*, *51*).

**Fig. 2.**
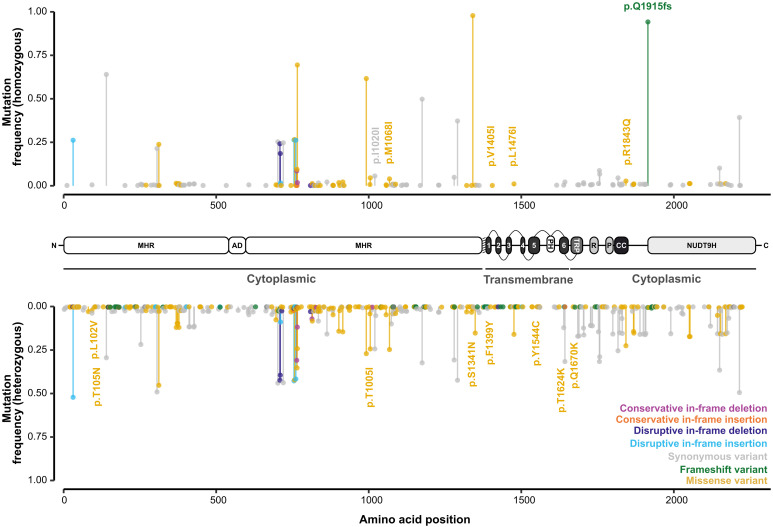
Genetic variation in the candidate mediator of praziquantel susceptibility, the transient potential receptor channel *Sm.*TRPM_PZQ_. Mutational frequency along the protein structure of *Sm.*TRPM_PZQ_ (*Smp_246790*). Frequencies of mutations are reported across 550 *S. mansoni* accessions (*y* axis), representing samples from Eastern Uganda (*n* = 17), Southern Uganda (*n* = 328), the Koome islands (*n* = 174), and Northern Tanzania (*n* = 31). *x*-axis values represent the location of the mutations on the protein, and bars (and terminal points) are colored by the predicted impact of each mutation. Structure of *Sm.*TRPM_PZQ_: N-terminal TRPM homology region (MHR) domain, ankyrin-like repeat domain (AD), pre-S1 helix (shaded), TM-spanning helices (1 to 6), pore helices (PH), TRP domain (TRP), rib helices (R), pole helices (P), coiled-coil (CC) region, and the COOH-terminal NUDT9H domain (NUDT9H).

**Fig. 3.**
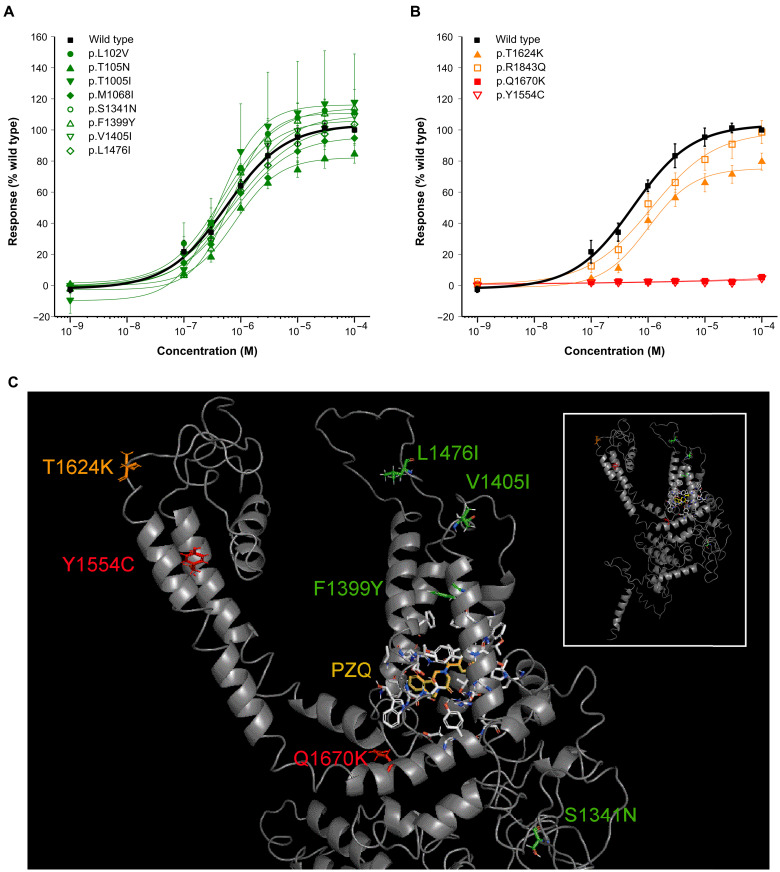
Functional profiling of variants of the transient potential receptor channel *Sm*.TRPM_PZQ_. Concentration-response relationships for the consensus *Sm.*TRPM_PZQ_ sequence compared with 12 Sm.TRPM_PZQ_ variants, which exhibit an average EC_50_ ± PZQ of (**A**) <1 μM (green) or (**B**) >1 μM (orange) to the presence or absence of praziquantel, respectively. Constructs for which no praziquantel-evoked activity was observed (Y1554C and Q1670K) are shown in (B) in red. Results represent the means ± SEM from at least three independent transfections. (**C**) Inset: Homology model of the transmembrane spanning region (residues 1100 to 1800) of an *Sm*.TRPM_PZQ_ monomer from (*40*). The enlarged view shows the location of the functionally profiled variants. Praziquantel and residues within 5 Å of the praziquantel binding poise (white) are shown at the base of the VSLD of the channel, with the S5 and S6 pore-forming helices to the left.

Consistent with previous genomic surveys (*43*, *45*), analysis of haplotype diversity using the integrated haplotype score (iHS) did not suggest that *Sm.*TRPM_PZQ_ was under positive selection in Lake Victoria *S. mansoni* populations (fig. S3 and tables S5 and S6). However, analysis of variation within *Sm.*TRPM_PZQ_ identified a high degree of variability, with predicted 501–amino acid changes across 437 of 2268 residues (Fig. 2), of which mutations at 178 of these residues were found only in single accessions (table S7). We generated point mutations to modify 12 *Sm*.TRPM_PZQ_ residues, on the basis of predicted deleterious likelihood, and used an in vitro Ca^2+^ reporter assay (*39*) to resolve their effect on praziquantel activity at *Sm*.TRPM_PZQ_. Eight of these mutants, all conservative amino acid substitutions, exhibited similar praziquantel sensitivity [range median effective concentration (EC_50_) = 0.49 to 0.96 μM] to the wild-type *Sm*.TRPM_PZQ_ channel {EC_50_ = 0.74 μM [standard error (SE) = 0.17]; Fig. 3A, table S8, and fig. S4}. Mutants p.T1624K and p.R1843Q exhibited decreased sensitivity to praziquantel [EC_50_ = 1.41 μM (SE = 0.23) and EC_50_ = 1.11 μM (SE = 0.08), respectively], and two mutants (p.Y1554C and p.Q1670K) caused a complete loss in praziquantel sensitivity (Fig. 3B, table S8, and fig. S5).

Seven of the 12 mutations mapped within an existing homology model for the transmembrane-spanning region of *Sm*.TRPM_PZQ_, enabling structural insight into these functional effects (Fig. 3C) (*40*). Four mutations had no impact on praziquantel action (p.V1405I, p.L1476I, p.S1341N, and p.F1399Y; Fig. 3A): p.V1405I and p.L1476I are found within extracellular loops of the channel, p.S1341N lies within the COOH-terminal cytoplasmic domain, and p.F1399Y, while within the VSLD of the channel that harbors the praziquantel binding site, is remote (∼10 Å away) from the praziquantel binding poise (Fig. 3C). Mutation p.T1624K (approximately twofold lower sensitivity; Fig. 3B) projects into the extracellular milieu before the start of S6 and may cause suboptimal orientation of the S6 helix. The other variant associated with lower sensitivity (p.R1843Q; Fig. 3B) lies outside the existing *Sm*.TRPM_PZQ_ homology model but is predicted to localize within the COOH-terminal coiled-coil region of the channel, which is involved in subunit interaction (*52*). Tetramer misfolding and lower channel expression would explain the decreased sensitivity of this variant.

The first of the two mutations that caused a complete loss of praziquantel sensitivity (Fig. 3B), p.Y1554C, is found in the S5 helix (Fig. 3C). This residue projects toward S6, where it is predicted to interact with a tyrosine residue (Y1636) in the S6 helix. This cysteine mutant would be expected to disrupt this interaction, which is likely important for the appropriate orientation of the helices that form the pore-forming domain. The second, p.Q1670K, introduces a positive charge within the intracellular TRP helix close to the bilayer interface. This is a critical region for channel gating, and the mutation likely affects the orientation of the TRP helix relative to the bilayer and its interaction with the S4/S5 loop of *Sm*.TRPM_PZQ_, which regulates TRPM channel activation (*53*). The deleterious effects of p.Y1554C and p.Q1670K are, therefore, consistent with the known importance of these regions for channel function (*40*).

Of the four mutations that decreased or eliminated praziquantel sensitivity, three (p.Y1554C, p.Q1670K, and p.T1624K) were only found in a heterozygous state in single accessions (table S10). The p.R1843Q variant, caused by the pos.2760344G>A variant, was more common, identified in 26% of accessions sampled from pre- or posttreatment populations (table S10; *n*_homozygotes_ = 120 of 519; *n*_heterozygotes_ = 15 of 519). The overall frequency of pos.2760344G>A was lower in posttreatment relative to pretreatment populations (freq_posttreatment_ = 0.208; freq_pretreatment_ = 0.287; χ^2^ = 2.183, df = 1, *P* = 0.1395); however, there was a slightly higher but nonsignificant frequency of homozygous genotypes in the posttreatment (*n* = 6 of 178; freq_homozygotes_ = 3.37%) compared with pretreatment (*n* = 9 of 341; freq_homozygotes_ = 2.64%) populations. We note that despite this small increase in frequency, accessions with this variant (and all other evaluated variants) did not form a distinct subpopulation (figs. S6 to S9). In addition to the mutations analyzed via targeted mutagenesis, we identified a second high-prevalence homozygous frameshift mutation, p.Q1915fs (pos.2760555dupC), in 94.0% of accessions. Park and colleagues (*40*) reported that truncation mutations (Δ1914) in the C-terminal NUDT9H domain do not affect TRP channel responsiveness to praziquantel.

Le Clec’h and colleagues (*41*) recently identified three putative marker variants associated with praziquantel resistance in selected laboratory lines. The first of these was a homozygous SNP predicted to result in a synonymous mutation (*Sm.*TRPM_PZQ_-2723187C; p.I1020l), which we identified in 29.3% of accessions sampled from pre- or posttreatment populations (table S10). This mutation was significantly higher in frequency (χ^2^ = 9.6651, df = 1, *P* = 0.001878) in posttreatment populations (37.07% of accessions; freq_heterozygotes_ = 28.65%; freq_homozygotes_ = 8.43%) compared to pretreatment populations (25.2% of accessions; freq_heterozygotes_ = 20.82%; freq_homozygotes_ = 4.40%). The second and third markers were 150-kb deletions adjacent to *Smp_246790* (∼1.22 Mb) and *Smp_345310* (∼3.18 to 3.33 Mb), respectively. *Smp_345310,* predicted to be a homolog of the transcription factor SOX-13, was strongly associated with praziquantel selection by Le Clec’h and colleagues (*41*) and was found among genes with elevated iHS, suggestive of being under selection (table S6). Structural variant genotyping did not identify the former variant in our accessions, but we did find a series of long homozygous deletions (69.9 to 215.0 kb) located between 3.02 and 3.36 Mb in 43.3% of genotyped accessions (*n* = 170 of 393 genotyped accessions; table S11 and fig. S10). However, the 150-kb deletion was not enriched in posttreatment populations, and neither the deletion nor p.I1020l clustered phylogenetically (fig. S9).

### Host infrapopulations reveal the extent of genetic relationships and evidence of treatment failure

Sampling and analysis of the parasite population within a single host (defined as the “infrapopulation”) at specific time points have the potential to identify treatment failures, which may be indicative of praziquantel resistance. However, these infrapopulations are thought to be highly heterogeneous (*37*, *54*, *55*), and previous genomic surveys of *S. mansoni* have included only small numbers of parasites from the same individuals (max *n* = 11) (*22*, *43*, *45*, *56*), reducing the likelihood of identifying related parasites. To provide a greater resolution of infrapopulation structure and evaluate the impact of praziquantel treatment, we analyzed pairwise kinship between 164 sequenced miracidia sampled pre– or post–praziquantel treatment from a total of three donors—Bb1 (*n =* 47 pretreatment and *n =* 42 posttreatment), Bu3 (*n =* 33 pretreatment and *n =* 19 posttreatment), and Bu1 (*n =* 2 pretreatment and *n =* 21 posttreatment)—and reanalyzed the 174 accessions from the survey of Vianney *et al.* (98 donors; *n* = 123 pretreatment and *n* = 51 posttreatment) (table S12) (*45*).

**Fig. 4.**
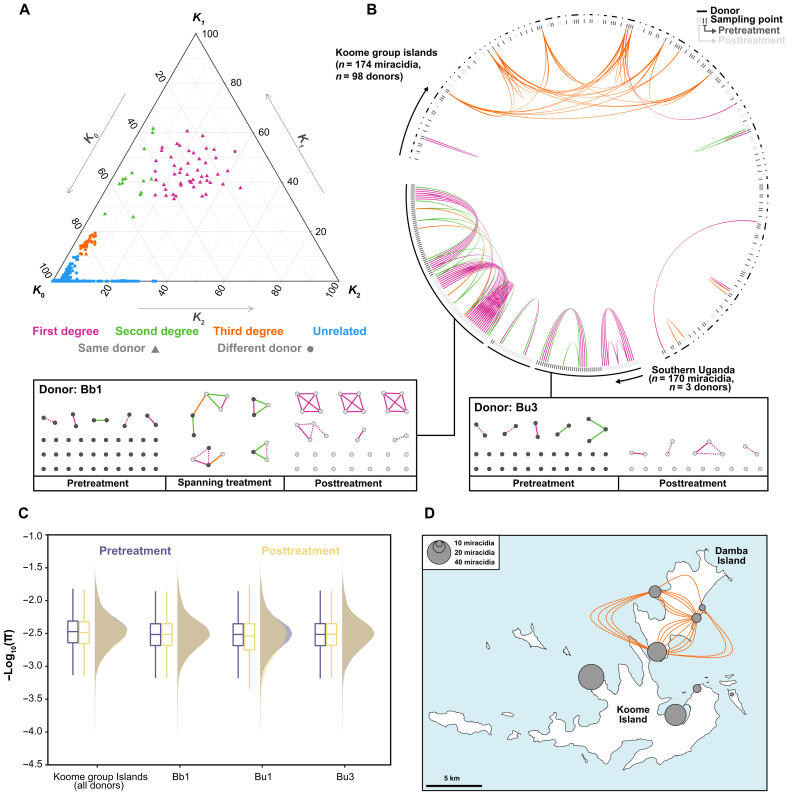
Relatedness between *S. mansoni* accessions. (**A**) Ternary plots of pairwise relationships between accessions using the three relatedness coefficients *K*_0_, *K*_1_, and *K*_2_. Each point represents a pairwise relationship, showing the probabilities that at a given locus, the two accessions shared zero-, one-, or two-allele identity by descent and if each accession was from the same (triangles) or different (circles) donor. Points are colored by the inferred relationship: first degree (pink), second degree (green), third degree (orange) or unrelated (blue). Only accessions from three Southern Ugandan donors and all Koome Island donors are shown. (**B**) Inferred relationships within and between donors. Outer lines represent each donor; within, individual accessions are colored by whether they were sampled before (dark gray) or after (light gray) praziquantel treatment. Colored lines represent the same pairwise relationships as (A). For donors Bb1 and Bu3, all relationships are shown below the circular plot and in boxes, grouped into pretreatment, posttreatment, and pre- and posttreatment (spanning treatment). Within the boxes, dotted lines represent relationships inferred by NgsRelate but found to have a lower degree or no relation by Sequoia. (**C**) The effect of praziquantel treatment on nucleotide diversity (π) was calculated for each donor infrapopulation (Bb1, Bu1, and Bu3) and all Koome group island samples. Nucleotide diversity was calculated in 5-kb nonoverlapping windows across each autosome for each pretreatment (purple) and posttreatment (yellow) population (including related samples). For all box plots, the central line indicates the median, and the top and bottom edges of the box indicate the 25th and 75th percentiles, respectively. Whisker lengths represent 1.5 times the interquartile range. (**D**) Identification of a circulating lineage of *S. mansoni* on Damba Island (the northernmost island of the Koome group islands). We identified a cluster of nine accessions, all with third-degree identical-by-descent relationships, that clustered phylogenetically with accessions from Lake Albert.

We identified 49 first-degree relationships, 13 second-degree relationships, and 34 third-degree relationships (Fig. 4A and table S13). Forty-seven of the 49 first-degree relationships were between accessions sampled from the same donor; the remaining two were between donors and have previously been identified as potentially mislabeled accessions (*45*). Estimates of the coefficient of inbreeding (*f*) using the condensed identity coefficients also revealed low levels of inbred relatedness (median *f* = 0.000227; range *f* = 1 × 10^−6^ to 0.35) in 18.8% of parents (table S12) (*57*). Within infrapopulations of donors Bb1, Bu1, and Bu3, we identified three first-degree relationships between pretreatment accessions and 23 between posttreatment accessions. Most of these were found in infrapopulations from Bb1, represented by three independent clusters of four accessions (Fig. 4, B and C). We also identified one first-degree and four second-degree relationships spanning treatment arms, suggesting bi- and uniparental survival, respectively. Both of the first-degree relatives were also homozygous for the *Sm.*TRPM_PZQ_-2723187C marker; however, none of the second-degree relatives had a homozygous or heterozygous copy. In addition, we did not identify any homo- or heterozygous variants of p.R1843Q in any of the first- or second-degree relatives spanning treatment. We also found no evidence of reduced nucleotide diversity in posttreatment populations (Fig. 4C) and low genetic differentiation between pre- and posttreatment populations (range mean *F*_ST_ = 1.24 × 10^−3^ to 1.75 × 10^−3^, fig. S11).

Examination of third-degree relationships within the dataset of Vianney and colleagues (*45*) identified a cluster of nine related accessions, all localized to Damba Island and found in all four of the sampled villages (Fig. 4, B and D). Phylogenetic analysis showed that all nine formed a distinct clade, with six accessions from Lake Albert (fig. S13), suggesting that these are derived from a recently imported lineage from Lake Albert that has recently dispersed across Damba Island.

## DISCUSSION

As efforts to eliminate schistosomiasis as a public health problem and interrupt transmission intensify over the next decade, the escalating use of praziquantel monotherapy for MDA is expected to result in increased selective pressures, altered transmission dynamics, and widespread reductions in schistosome populations, especially across sub-Saharan Africa (*1*, *9*). The consequences of such large-scale changes in schistosome populations are unclear, but they provide a strong incentive for genomic surveillance to detect and monitor them. In this study, we have, to our knowledge, assembled the most comprehensive collection of *Schistosoma* whole-genome sequencing data to date, including samples from across eight countries and two major foci of infection.

Praziquantel resistance represents a credible threat to schistosomiasis control if it were to establish in endemic populations. Despite more than 50 years of use, *Sm.*TRPM_PZQ_ has only recently been identified as a direct target of praziquantel and a genetic determinant of reduced praziquantel susceptibility (*40*, *41*). We identified extensive, low-frequency variation within this gene and used this variation database to inform the targeted mutagenesis of 12 residues, focusing on mutations at potential key residues on the basis of (i) their location within the transmembrane spanning region of *Sm.*TRPM_PZQ_ or (ii) proximity to known null *Sm.*TRPM_PZQ_ mutants previously identified in the laboratory. For (i), the transmembrane regions of TRPM channels are highly conserved through vertebrate evolution with high levels of sequence conservation in the VSLD [transmembrane 1 (TM1) to TM4], the pore domain (TM5 and TM6), and the TRP helix. This region of the channel exhibits higher constraint and lower tolerance for variation such that SNPs found in this region are more likely to be deleterious to praziquantel action. For (ii), the natural variants L102V and T105N are found in the NH_2_-terminal region of *Sm.*TRPM_PZQ_, close to laboratory-made mutants F107A and G108A, which are deleterious to channel function. We identified four mutations in our population genetic analyses that reduced or ablated channel responsiveness to praziquantel functional genetic in vitro assays. While only one (p.R1843Q) was present in more than a single accession, all represent standing variation for praziquantel resistance in endemic populations. Both p.R1843Q and p.I1020l [the latter being a marker for praziquantel resistance in laboratory-passaged lines (*41*)] variants were present in multiple accessions, indicating potential variability in praziquantel efficacy in these populations. Both mutations were also more prevalent in posttreatment parasite populations than their pretreatment counterparts. However, our analyses and others (*35*, *37*, *43*) have shown that posttreatment populations do not typically represent subpopulations of pretreatment parasites, which may instead result from reinfection or undersampling. The increased prevalence of these mutations is thus likely unrelated to praziquantel administration. It is clear, nevertheless, that despite the asymmetrical per-host sampling of this study, these variants remain widespread in parasite populations sampled from a wide range of hosts, showing that they are prevalent across Lake Victoria. Further work should now be undertaken to determine the extent to which the mutations we identified affect the in vivo efficacy of praziquantel and parasite fitness. Profiling these specific SNPs in the context of other changes within *Sm.*TRPM_PZQ_ in the same samples, reflecting broader allelic variation, is also necessary.

Our analyses have demonstrated how genomic surveillance of parasites can help identify variants contributing to reduced praziquantel efficacy in endemic populations and provide structural and functional insights into *Sm.*TRPM_PZQ_, thereby improving the understanding of praziquantel efficacy in schistosomes. While we only characterized a small proportion of the total variation we observed, our results survey genetic variation within endemic regions where praziquantel has been extensively used. These data provide candidate variants for functional investigations and serve as a database of variation for retrospective surveys once further resistance-conferring mutations are identified.

Targeted mutagenesis identified four mutations in endemic populations that reduced channel sensitivity to praziquantel, indicating standing variation for resistance. While we did identify both standing variation for future resistance evolution and instances of treatment failures here, the apparent lack of obvious high-frequency resistance-conferring mutations is encouraging in terms of reaching the WHO elimination targets, as these populations have been under long-term MDA pressure. The variable coverage of MDA may have allowed substantial refugia populations to exist in untreated humans, snails, and animal reservoirs. Refugia would reduce the selection pressure for resistance, an approach that has been deliberately used in veterinary systems (*58*–*60*). Alternatively, reduced praziquantel susceptibility might be linked to regulatory changes in expression or RNA splicing (*41*), neither of which would be identified by our analyses. Considering how, recently, *Sm.*TRPM_PZQ_ was recognized as a mediator of praziquantel sensitivity, future functional investigations will likely reveal additional relevant variants.

Despite involving a small number of donors, our analyses of host infrapopulation dynamics represent the most thorough genomic characterization of a host infrapopulation of any parasitic helminth. Our analyses found that host infrapopulations are exceptionally diverse, consistent with human autopsy studies and genetic surveys, which have found that individual worm burdens range from one to hundreds or thousands of worm pairs per individual in low- and high-endemicity regions, respectively (*61*, *62*). Despite this, we found evidence of sibling relationships in both treatment arms, with indications of a higher degree of posttreatment relatedness. We also found evidence of posttreatment parental survival, suggesting either ineffective treatment or higher praziquantel tolerance within these populations. However, the limited number of these accessions limited any formal analyses of the possible genetic basis of reduced praziquantel efficacy. Consistent with previous genetic studies, these represented only a small proportion of the overall posttreatment population (*37*), suggesting that most posttreatment samples reflect infection with additional parasites and/or parasites missed in pretreatment sampling.

To conclude, we have characterized endemic *S. mansoni* populations at multiple spatial scales ranging from comparisons across major foci of infection to individual parasite infrapopulations. Our variation analysis within a candidate praziquantel resistance locus enabled us to identify multiple novel resistance-conferring mutations. Although candidate resistance loci have not yet risen to high frequencies in the studied populations, they do demonstrate standing variation for praziquantel resistance in endemic populations undergoing MDA, potentially akin to the standing variation observed for oxamniquine before resistance emerging and rendering it no longer useful as an anti–*S. mansoni* drug. Thus, monitoring for phenotypic and genotypic evidence of praziquantel resistance needs to be considered if praziquantel is to remain effective for *S. mansoni*, and all *Schistosoma* species of humans and livestock, control. Our analyses also provide a resource for retrospective or confirmatory support of functional analyses of this recently identified locus. Last, our extensive sequencing of host infrapopulations provided an in-depth genomic characterization of parasite diversity and its impact on praziquantel treatment. Overall, our study summarizes *S. mansoni* genomic diversity and represents a resource to assess the efficacy of current interventions and guide future treatment strategies.

## MATERIALS AND METHODS

The data analyzed here incorporated whole-genome accessions from published datasets from eight countries and 205 new *Schistosoma* sample data (Fig. 1A, Table 1, and table S1). The sections below describe the origin, collection, and ethical approval of samples, focusing only on the parasite samples sequenced for this study and not previously published accessions.

### Collection and ethical approval for the sampling of Ugandan miracidia

The collection of all Ugandan miracidial samples was undertaken as part of the monitoring, evaluation, and disease control activities conducted by the Vector Control Division of the Ministry of Health (Uganda), the Schistosomiasis Control Initiative and Imperial College London. All methods and data collection were approved by the Uganda National Council for Science and Technology (Memorandum of Understanding: sections 1.4, 1.5, and 1.6) and the Imperial College Research Ethics Committee (EC no. 03.36; R&D no. 03/SB/033E). The Head of the Vector Control Division informed local district officials, and the headteachers of each school were informed about the study and requested to provide informed consent to allow sampling to be performed within the school. Parents of the children were informed of the study through school meetings, where they were provided with detailed information regarding the purpose of the study, and technical staff was present to answer questions. Parents were requested to provide informed consent for their children to participate in the study, and in addition, any children aged 10 years or older were asked to give informed consent after receiving complete information about the study. Participation was voluntary, access to treatment was not contingent on study participation, and children could withdraw from the study at any time.

Stool samples were collected from each child, and duplicate Kato-Katz thick smears were conducted (*63*). A Pitchford-Visser funnel was used to wash and filter parasite eggs from the remaining stool sample, and the filtrate was stored overnight (*64*). Miracidia were hatched the following day and were transferred into two sequential dishes of nuclease-free water to dilute bacterial contaminants before being individually fixed onto Whatman FTA-indicating classic cards (*65*, *66*). Between 1 and 3 days following testing, children with evidence of parasitic infection were treated with praziquantel (40 mg/kg) for schistosomiasis and albendazole (400 mg) for soil-transmitted helminths. Children were retested 25 to 27 days following treatment, i.e., at a time point before any subsequent *S. mansoni* infections could establish patency, but after which any potential viable eggs laid just before praziquantel treatment are unlikely to continue to be expelled. In cases of incomplete *S. mansoni* clearance, miracidia were sampled again, and treatment was readministered.

### Collection and ethical approval for the sampling of Ugandan *S. mansoni* cercariae

The FTA-preserved Ugandan *S. mansoni* cercariae were collected as part of snail collection surveys conducted between 2007 and 2010 as part of the EU-CONTRAST program, a consortium of European and African researchers (CONTRAST EU/INCO.Dev contract no. 032203) in association with the Schistosomiasis Control Initiative (*67*), and provided via the Schistosomiasis Collection at the Natural History Museum [SCAN; (*65*, *68*)]. As for the miracidial sampling above, all methods for cercarial sampling were approved by the Uganda National Council for Science and Technology (Memorandum of Understanding: sections 1.4, 1.5, and 1.6) and the Imperial College Research Ethics Committee (ICREC no. 03.36; R&D no. 03/SB/033E).

### Collection and ethical approval for the sampling of Tanzanian *S. mansoni* cercariae

Tanzanian *S. mansoni* cercariae were originally collected as part of snail collection surveys undertaken in the Mwanza and Geita regions of Northern Tanzania between January 2012 and December 2015 (*69*) as part of the Schistosomiasis Consortium for Operational Research and Evaluation (SCORE) snail project (*70*) and archived within SCAN. Ethical approvals were provided by the Imperial College Research Ethics Committee Imperial College London, UK, in combination with the ongoing Schistosomiasis Control Initiative activities (ICREC no. 03.36; R&D no. 03/SB/033E); the Ethical Review Board of National Institute of Medical Research (NIMR) in Tanzania; and the Institutional Review Board of the University of Georgia in Athens, GA (project no. 2012-10138-0). Survey sites were picked on the basis of their proximity (within 5 to 15 m) to schools and were identified on the basis of local information about water activities (bathing, fishing, and water collection) (*70*). *Biomphalaria* spp. snails were collected by scooping with handheld metal sieve scoops or by dredging with a metal dredge dropped from a boat and dragged 10 m back to shore. Scooped/dredged snails were hand collected with forceps and placed into collection jars. All snails were placed in 24-well enzyme-linked immunosorbent assay plates under direct light for at least 4 hours to induce shedding, and cercariae from each shedding snail were individually collected in 3 μl of water using a Gilson pipette and fixed on Whatman FTA cards (*65*, *66*). As *S. rodhaini* and *S. rodhaini*:*S. mansoni* hybrids have also been documented to be shed from *Biomphalaria* spp. in Tanzania (*47*), cercariae were also sequenced by COX-1 (cyclooxygenase 1) and internal transcribed spacer (ITS), as described by Standley *et al.* (*71*), to confirm that the cercarial samples included were *S. mansoni*.

### Origin of the Senegalese *S. mansoni* laboratory (adult worm) sample

Both *S. mansoni* Senegalese isolates (FS0001 and FS0002) originated from ∼30 *Biomphalaria pfeifferi* with patent *S. mansoni* infections found in the Western principal irrigation canal in the Ndiengue District of Richard Toll (*72*). The strain was originally sampled in 1993; maintained at the School of Biological Science, University of Wales (Bangor, United Kingdom); and subsequently in the helminGuard laboratory (Süelfeld, Germany) (*73*).

### Origins of the *S. rodhaini* (adult worms and cercariae) samples

The adult *S. rodhaini* adult worm sample (RZ0001) was provided by SCAN from a laboratory-passaged *S. rodhaini* strain originally isolated from infected *Biomphalaria* snails collected in Burundi in 2000. The sample used here represented the sixth passage through laboratory *Biomphalaria glabrata* and *Mus musculus* under the Home Office project license numbers 70/4687 (before 2003) and 70/5935 (2003 to 2008) (*65*).

The two *S. rodhaini* cercariae analyzed here originated from the Mwanza region of Tanzania. They were collected as part of the SCORE xenomonitoring project and provided by SCAN. As for the *S. mansoni* cercariae from this region in Tanzania above, ethical approvals were provided by the Imperial College Research Ethics Committee Imperial College London, UK, in combination with the ongoing Schistosomiasis Control Initiative activities (ICREC no. 03.36; R&D no. 03/SB/033E); the Ethical Review Board of National Institute of Medical Research (NIMR) in Tanzania; and the Institutional Review Board of the University of Georgia in Athens, GA (project no. 2012-10138-0). Species identification was confirmed by Sanger sequence analysis of a partial region of the *cox1* gene, the ITS1+2 recombinant DNA region, and a partial region of the 18*S* recombinant DNA region using the methods described by Pennance *et al.* (*74*).

### DNA extraction and sequencing

The DNA from individual miracidia and cercariae was isolated from the Whatman FTA cards using methods described in (*75*, *76*). A 2-mm Harris micropunch was used to punch out the FTA disc containing the DNA. Within a well of a 96-well polymerase chain reaction plate, individual samples on individual punches were lysed in 30 μl of the following buffer: 30 mM tris-HCl, pH 8.0 (Sigma-Aldrich); 0.5% Tween 20 (Sigma-Aldrich); 0.5% NP-40/IGEPAL CA-630 (Sigma-Aldrich); and protease reagent (1.25 μg/ml; Qiagen; cat. no. 19155). Punches were incubated at 50°C for 1 hour and then heated to 75°C for 30 min. DNA was extracted from adult worms using the Qiagen MagAttract HMW kit (PN-67653) following the manufacturer’s instructions. The DNA from the two individual Senegalese adult worms was extracted using the Qiagen MagAttract HMW kit (PN-67653) following the manufacturer’s instructions.

Library preparation was performed using a low-input enzymatic fragmentation-based library preparation method (*77*). For each sample, 20 μl of lysate (or extracted DNA) was mixed with 50 μl of tris-EDTA (TE) buffer (Ambion, 10 mM tris-HCl and 1 mM EDTA) and 50 μl of Ampure XP beads, followed by a 5-min binding reaction at room temperature. Magnetic bead separation was used to separate genomic DNA, which was then washed twice with 75% ethanol. Beads were resuspended in 26 μl of TE buffer. To perform DNA fragmentation and A-tailing, each sample was immediately mixed with 7 μl of 5× Ultra II FS buffer and 2 μl of Ultra II FS enzyme and incubated on a thermal cycler for 12 min at 37°C, followed by 30 min at 65°C. Adaptor-ligated libraries were prepared by adding 30 μl of ligation mix, 1 μl of ligation enhancer (New England BioLabs), 0.9 μl of nuclease-free water (Ambion), and 0.1 μl of duplexed adapters to each well, followed by incubation for 20 min at 20°C. Libraries were purified and eluted by adding 65 μl of Ampure XP beads and 65 μl of TE buffer. Libraries were amplified by adding 25 μl of KAPA HiFi HotStart ReadyMix (KAPA Biosystems) and 1 μl of PE1.0 primer to 21.5 μl of the library. Each sample was thermal-cycled as follows: 98°C for 5 min and then 12 cycles of 98°C for 30 s, 65°C for 30 s, 72°C for 1 min, and lastly, 72°C for 5 min. Ampure beads were used to purify amplified libraries using a 0.7:1 volumetric ratio of beads to library. Each library was then eluted into 25 μl of nuclease-free water. Library concentrations were adjusted to 2.4 nM and pooled, followed by sequencing on the Illumina NovaSeq 6000 using 150–base pair paired-end chemistry.

### Variant discovery and annotation

In addition to the whole-genome sequencing of samples described above, sequence data for 407 *S. mansoni* accessions (*43*–*45*) and one *S. rodhaini* accession (*44*) were included in this study. Raw sequencing reads from all 682 accessions were trimmed using BBDuk (https://github.com/bbushnell/BBTools) to remove low-quality bases and adapter sequences. Trimmed sequence reads were aligned to the *S. mansoni* (SM_V9, WormBase ParaSite version 16) (*78*) reference genome using BWA mem (version 0.7.17) (*79*). Polymerase chain reaction duplicates were marked using PicardTools MarkDuplicates (as part of GATK version 4.2.0.0) (*80*). Variant calling was performed per sample using GATK HaplotypeCaller (version 4.2.0.0) in gVCF (genomic variant call format) mode, retaining both variant and invariant sites. Individual gVCFs were merged using GATK CombineGVCFs, and joint-call cohort genotyping was performed using GATK GenotypeGVCFs. Variant sites with only SNPs were separated from indels and mixed sites (variant sites that had both SNPs and indels called) using GATK SelectVariants. GATK VariantFiltration was used to filter both groups independently. SNPs were retained if they met the following criteria: QD ≥ 2.0, FS ≤ 60.0, MQ ≥ 40.0, MQRankSum ≥ −12.5, ReadPosRankSum ≥ −8.0, and SOR ≤ 3.0. Variant sites containing indels or mixed sites were retained if they met the following criteria: QD ≥ 2.0, FS ≤ 200.0, ReadPosRankSum ≥ −20.0, and SOR ≤ 10.0.

VCFtools (version 0.1.15) was used to exclude accessions with a high rate of variant-site missingness (missing genotype called at >5% of sites) and subsequently to remove sites where >10% of accessions had a missing genotype (*81*). This formed the primary VCF file used for almost all analyses. For analyses of nucleotide diversity and fixation index (*F*_ST_), we produced a second filtered VCF file. We used VCFtools to filter both variant and invariant sites with >80% missing variants; enforced a minimum mean read depth of 5 and a maximum mean read depth of 500, removing variant sites found to be significantly out of Hardy-Weinberg equilibrium (*P* < 0.001); and only retained SNPs. Functional annotation of SNPs and indels in the primary VCF file was performed using SnpEff (version 5.0e) (*82*) with gene annotations (version 9) downloaded from WormBase ParaSite version 17 (*83*). Repetitive elements in the *S. mansoni* assembly were annotated using RepeatModeler and RepeatMasker (*84*).

### Depth of coverage

For each sample, the depth of read coverage was calculated in 2-kb windows across each chromosome using bedtools coverage (version 2.30.0) (*85*).

### Sample relatedness

We estimated pairwise relatedness between accessions from the same endemic regions. We subsampled to autosomal SNPs only and ran NgsRelate (*86*) with default parameters. As an additional line of evidence, we first used PLINK (version 2.0) (*87*) to exclude SNPs in strong linkage disequilibrium. The genome was then scanned in sliding windows of 50 SNPs, increasing in steps of 10 SNPs, and SNPs within windows with squared correlation coefficients >0.2 were removed. Using this filtered SNP subset, we performed pedigree reconstruction with Sequoia (*88*). Pairwise relationships were first classified on the basis of the coefficient of kinship score (θ) calculated by NgsRelate. NgsRelate was first run on all 570 *S. mansoni* samples to identify relationships between datasets or populations. After confirming that these relationships did not occur, NgsRelate was rerun on individual populations from Southern Uganda, Northern Tanzania, and the Koome Islands. In addition, samples from Berger *et al.* (*43*) were excluded from these analyses because of the high inbreeding coefficients in this dataset. Pairwise relationships with θ > 0.354 were classified as monozygotic twins, those with 0.354 > θ ≥ 0.177 were classified as first-degree relatives, those with 0.177 > θ ≥ 0.0884 were classified as second-degree relatives, and those with 0.0884 > θ ≥ 0.0442 were classified as third-degree relatives. In addition, first-, second-, and third-degree relationships were only considered valid if the maximum likelihood estimate of sharing one identity-by-descent allele (*K*_1_) was greater than 0.05. Instances where both NgsRelate and Sequoia identified the same first-degree relationships were designated as “high-confidence” relationships. GGtern was used to plot all three maximum likelihood estimates of sharing 0, 1, or 2 identity-by-descent alleles (*K*_0_, *K*_1_, and *K*_2_), and Circos (*89*) and Gephi (*90*) were used to visualize pairwise relationships between different donors.

### Population genomic structure and diversity

We removed variants with minor allele frequencies <0.05 and excluded all variants on the Z chromosome, W chromosome, and mitochondrial genome. We then removed all variants found within repetitive regions identified by RepeatMasker and lastly removed variants in strong linkage disequilibrium, as above. Principal components analysis was performed with the remaining 188,923 autosomal SNPs using PLINK. Admixture analyses were performed using ADMIXTURE (*91*) with *K* values (number of hypothetical ancestral populations) ranging from 1 to 20, 10-fold cross-validation, and standard error estimation with 250 bootstraps. The lowest cross-validation error value was found for *K* = 5 (fig. S13). In addition, principal components analysis was repeated, as described above, except using only 505 unrelated *S. mansoni* accessions using 214,445 autosomal SNPs.

We used publicly available scripts to convert all 188,923 autosomal SNPs into Phylip format (vcf2phylip.py; https://github.com/edgardomortiz/vcf2phylip/) and remove invariant sites (ascbias.py; https://github.com/btmartin721/raxml_ascbias/). Phylogenomic inference was performed using IQ-TREE (*92*) using the best-fit substitution model with ascertainment-bias correction selected by ModelFinder (GTR+F+ASC+R10) and 1000 ultrafast bootstraps. The resulting phylogeny was visualized using ggtree (*93*).

pixy (version 1.2.3.beta1) (*94*) was used to calculate autosomal nucleotide diversity (π) and the fixation index (*F*_ST_) in 5-kb nonoverlapping, sliding windows for each population using the secondary (mixed variant and invariant sites) VCF. Negative *F*_ST_ values were corrected to 0 before calculating genome-wide median values. Watterson’s estimator (Θ) was calculated using Scikit-allel (*95*). The effective population size (*N*_e_) was calculated using a per-generation mutation rate (μ) of 8.1 × 10^−9^ (*44*) and the following equation

$$N_{e}=\Theta /4\mu$$

The coefficient of inbreeding (*F*) was calculated using VCFtools to assess per-sample homozygosity. Comparisons of samples within and between study sites can be found in fig. S14 (A to C).

The iHS was calculated for each subpopulation using Selscan (version 1.2.0a) (*96*), as previously described (*43*). Median iHS values were calculated across all variants in 5-kb nonoverlapping windows along the seven *S. mansoni* autosomes. Windows with fewer than 10 variants per 5-kb window were removed. Windows with elevated iHS scores (|iHS| > 2) within 50 kb of another window were grouped into continuous regions of selection.

### Impact of praziquantel treatment on population genomic diversity

We used pixy (version 1.2.3.beta1) to calculate autosomal nucleotide diversity (π) in 5-kb nonoverlapping, sliding windows for each pre- and posttreatment population using the secondary (mixed variant and invariant sites) VCF and including related accessions (*94*).

### Structural variant genotyping and annotation

Structural variants for each sample were detected using LUMPY (version 0.2.13) and genotyped using SVTyper (version 0.7.0), as implemented in the Smoove (version 0.2.7) pipeline (https://github.com/brentp/smoove) (*97*, *98*). Using this pipeline, we first performed single-sample calling using the BWA-aligned reads to the *S. mansoni* reference genome. This was followed by merging called variants and sample-wise regenotyping across all 570 *S. mansoni* accessions. Structural variants intersecting coding regions were annotated using the reference GFF file. We then filtered variants on the basis of the Smoove author’s recommendations, excluding heterozygous calls with MSHQ (mean Smoove heterozygote quality) >= 3, deletions with DHFFC (duphold flank fold change) >= 0.7, and duplications with DHFFC <= 1.25. Because of inconsistent coverage and whole-genome amplification of the original libraries, all accessions from Berger *et al.* (*43*) were excluded from structural variant analyses after genotyping.

### Inference of demographic history

We ran SMC++ (version 1.15.2) (*99*) on each autosome using a per-generation mutation rate of 8.1 × 10^−9^ and a generation time of 85 days. For Southern Ugandan, Koome Island, Northern Tanzanian, and Eastern Ugandan populations, we randomly subset them down to *n* = 12 unrelated accessions, providing replicates where populations had more than 24 accessions. For all other populations, only single accessions were analyzed.

### Screening for recent admixture

Analysis of laboratory-passaged strains also identified a single probable *S. mansoni*-*S. rodhaini* hybrid (RZ0001), which displayed high heterozygosity, intermediate admixture proportions, and intermediate phylogenetic positioning, consistent with an early-generation (F_1_ to F_3_) hybrid; this sample was excluded from further analyses (fig. S15 and table S2) (*100*, *101*).

### Analysis of candidate praziquantel resistance alleles

We inspected SnpEff-annotated variants within the candidate praziquantel resistance gene (*Smp_24790*), focusing on isoform 5 (*Smp_246790.5* in the version 9 annotation), the only full-length isoform containing the predicted binding site. Frequencies of functionally impactful mutations were calculated using only Lake Victoria accessions. The previously produced maximum-likelihood phylogeny was annotated to highlight accessions containing functionally impactful mutations. We excluded a single indel, p.Met1fs, which was a frameshift variant and a short repeat from the analysis; for completeness, information on this variant is still reported in tables S7, S9, and S10.

### Ca^2+^ reporter assays

Ca^2+^ imaging assays were performed using a fluorescence imaging plate reader (FLIPR^TETRA^, Molecular Devices). Human embryonic kidney 293 cells (naïve or transfected with specific *Sm.*TRPM_PZQ_ variants) were seeded (20,000 cells per well) in a black-walled, clear-bottom poly-d-lysine–coated 384-well plate (Corning) in Dulbecco’s modified Eagle’s medium growth media supplemented with 10% fetal bovine serum. This medium was removed after 24 hours, and the cells were loaded with a fluorescent Ca^2+^ indicator (Fluo-4 NW dye, Invitrogen) by incubation (20 μl per well, 1 hour at 37°C) in Hanks’ balanced salt solution assay buffer containing probenecid (2.5 mM) and Hepes (20 mM). Dilutions of praziquantel (Sigma-Aldrich) were prepared in the same buffer (1 nM to 100 μM, final concentration), without probenecid or dye, in flat-bottom 384-well plates (Greiner Bio-One, Germany). The Ca^2+^ reporter assay was performed at room temperature by monitoring fluorescence (raw fluorescence units) before (basal, 20 s) and after the addition of praziquantel (an additional 250 s). For quantitative analyses, peak fluorescence in each well was normalized to the maximal response of the reference channel sequence, and concentration-response curves were plotted using the sigmoidal dose-response function in Origin.
