# Supplementary material for: Extensive parasite transmission and variation in a functional receptor associated with drug resistance in endemic Schistosoma mansoni
Source: Sci Adv. 2026 Jul 17;12(29):eadt3721. doi: 10.1126/sciadv.adt3721 (PMC13378547; doi:10.1126/sciadv.adt3721)
Supplement: Supplementary file 1 — Figs. S1 to S15 Legends for tables S1 to S14 [file sciadv.adt3721_sm.pdf]

Science Advances
AAAS

Supplementary Materials for

Extensive parasite transmission and variation in a functional receptor associated with drug resistance in endemic *Schistosoma mansoni*

Duncan J. Berger *et al.*

Corresponding author: Duncan J. Berger, duncan.berger@bath.edu;
Matthew Berriman, matt.berriman@glasgow.ac.uk; Jonathan S. Marchant, jmarchant@mcw.edu;
Stephen R. Doyle, sd21@sanger.ac.uk; Joanne P. Webster, jowebster@rvc.ac.uk

Sci. Adv. **12**, eadt3721 (2026)
DOI: 10.1126/sciadv.adt3721

The PDF file includes:

Figs. S1 to S15
Legends for tables S1 to S14

Other Supplementary Material for this manuscript includes the following:

Tables S1 to S14

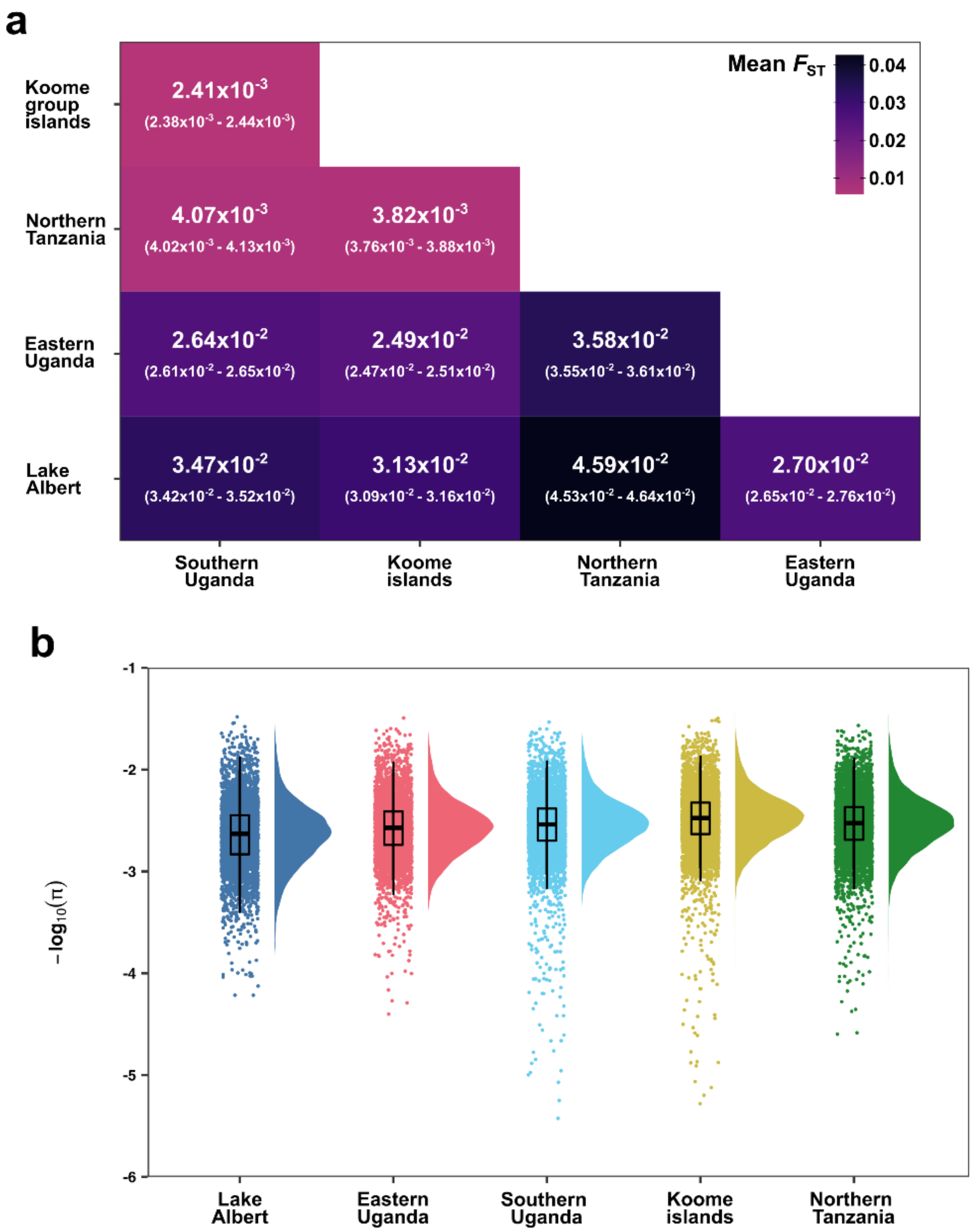

Figure S1. Diversity and differentiation of Lake Victoria *Schistosoma mansoni* populations. **a)** Pairwise comparisons of fixation index (F_{ST}) between each population with greater than two unrelated accessions representing Lake Albert (n = 3 accessions), Eastern Uganda (n = 17), Southern Uganda (n = 287), Koome group islands (n = 159) and Northern Tanzania (n = 30). F_{ST} was calculated using autosomal variants in non-overlapping 5 kb windows. Mean values for each comparison are shown in bold, and numbers in parentheses represent the 95% bootstrap confidence intervals around the mean. **b).** Autosomal nucleotide diversity (π) values are calculated as the mean of non-overlapping 5 kb windows for each population described in d). For all boxplots, the central line indicates the median, the top and bottom edges of the box indicate the 25th and 75th percentiles, respectively. The maximum whisker lengths are specified as 1.5 times the interquartile range.

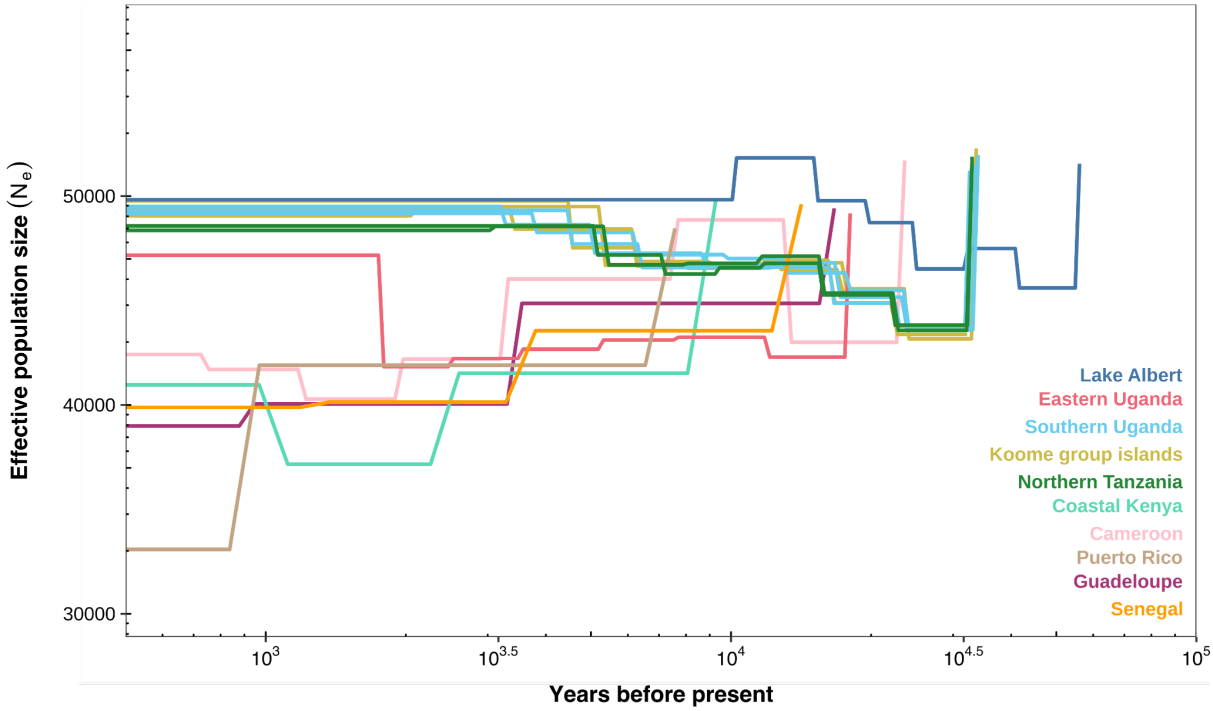

Figure S2. Inference of demographic history using SMC++ for *Schistosoma mansoni* subpopulations.

SMC++ was run on each autosome using a per-generation mutation rate of $8.1x10^{-9}$ and a generation time of 85 days. We randomly subset the populations to *n* = 12 unrelated accessions for the Eastern Ugandan, Southern Ugandan, Koome group island, and Northern Tanzanian populations, providing replicates when populations had more than 24 accessions. For all other populations, lines represent single accessions.

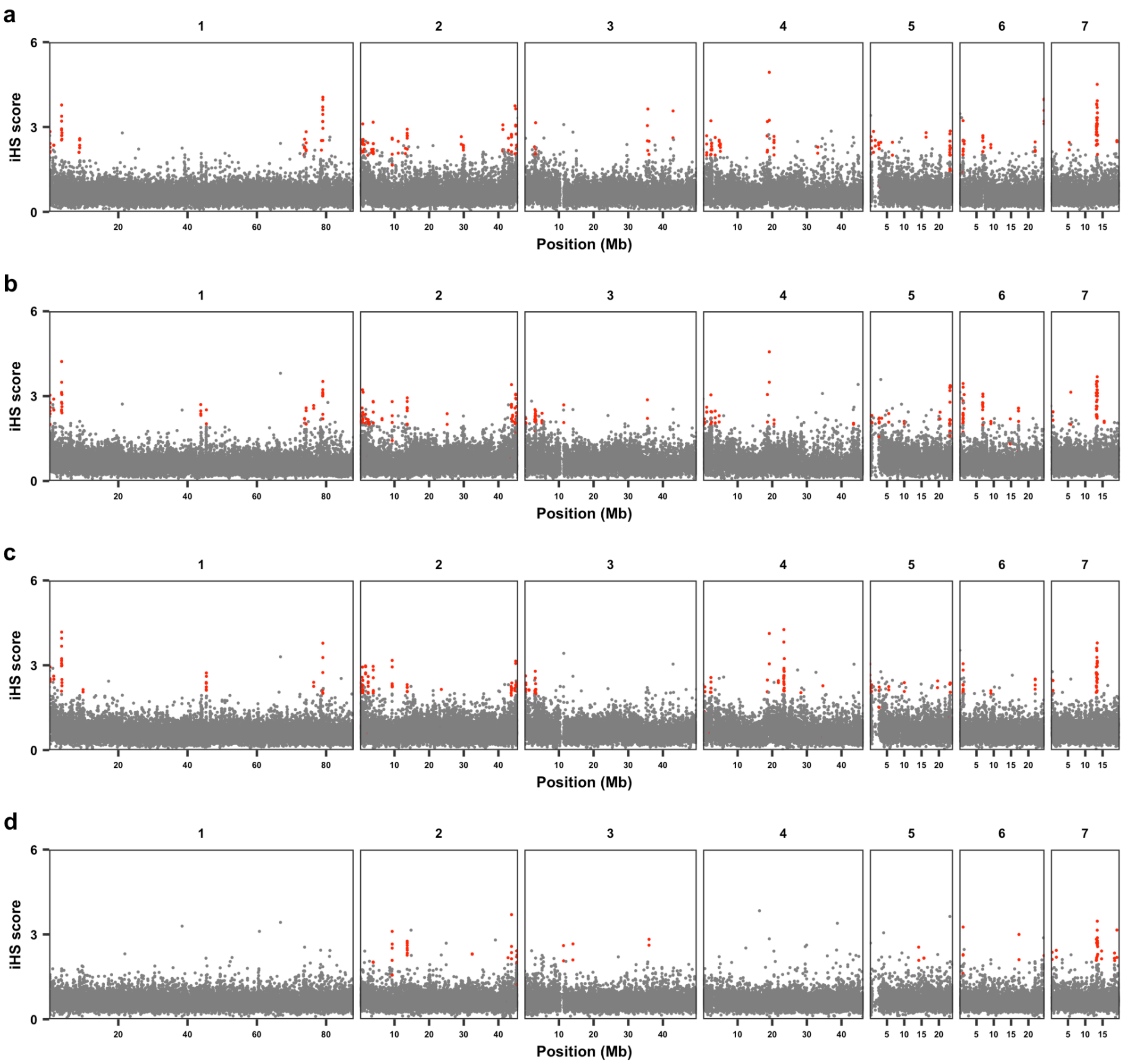

Figure S3. Genome-wide integrated haplotype scores (iHS) were calculated independently using unrelated accessions from four populations.

a) Southern Uganda (*n* = 287), **b)** Koome group islands (*n* = 159), **c)** Northern Tanzania (*n* = 30) and **d)** Eastern Uganda (*n* = 17). Points represent median |iHS| values of all variants in 5 kb non-overlapping windows along the seven *S. mansoni* autosomes (grey). Windows with elevated iHS scores (|iHS|>2) within 50 kb of another window were grouped into continuous regions of selection; windows within those regions are shown in red.

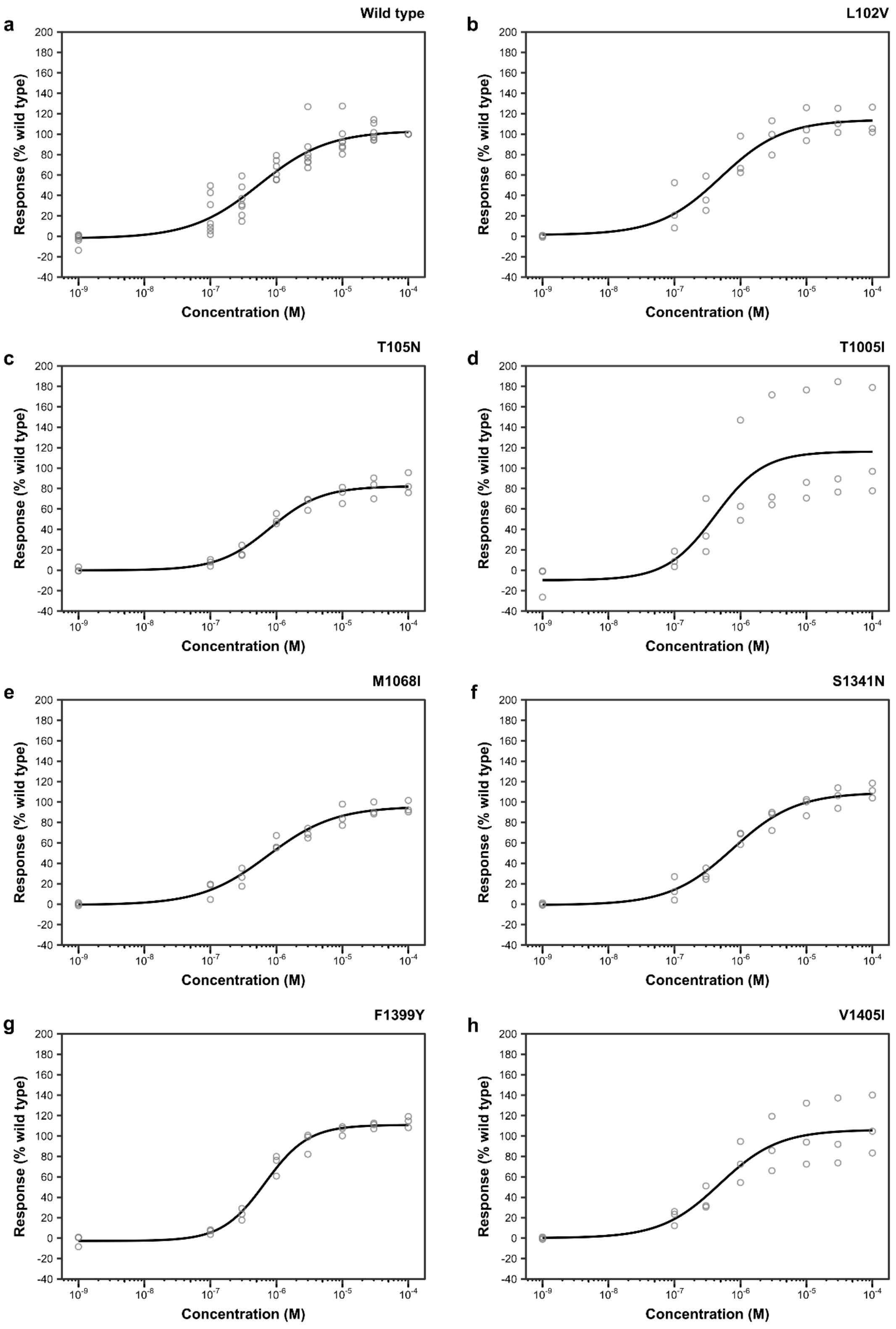

Figure S4. Functional profiling of *Sm*.TRPM$_{PZQ}$ variants.
Concentration-response relationships for the consensus *Sm*.TRPM$_{PZQ}$ sequence compared with seven *Sm*.TRPM$_{PZQ}$ variants. **a)** Wild-type, **b)** L102V, **c)** T105N, **d)** T1005I, **e)** M1068I, **f)** S1341N, **g)** F1399Y, **h)** V1405I. Results represent mean response relative to **a)** wild type from at least three independent transfections (points). Lines represent the fitted dose-response model using the four-parameter log-logistic function implemented in the drc package.

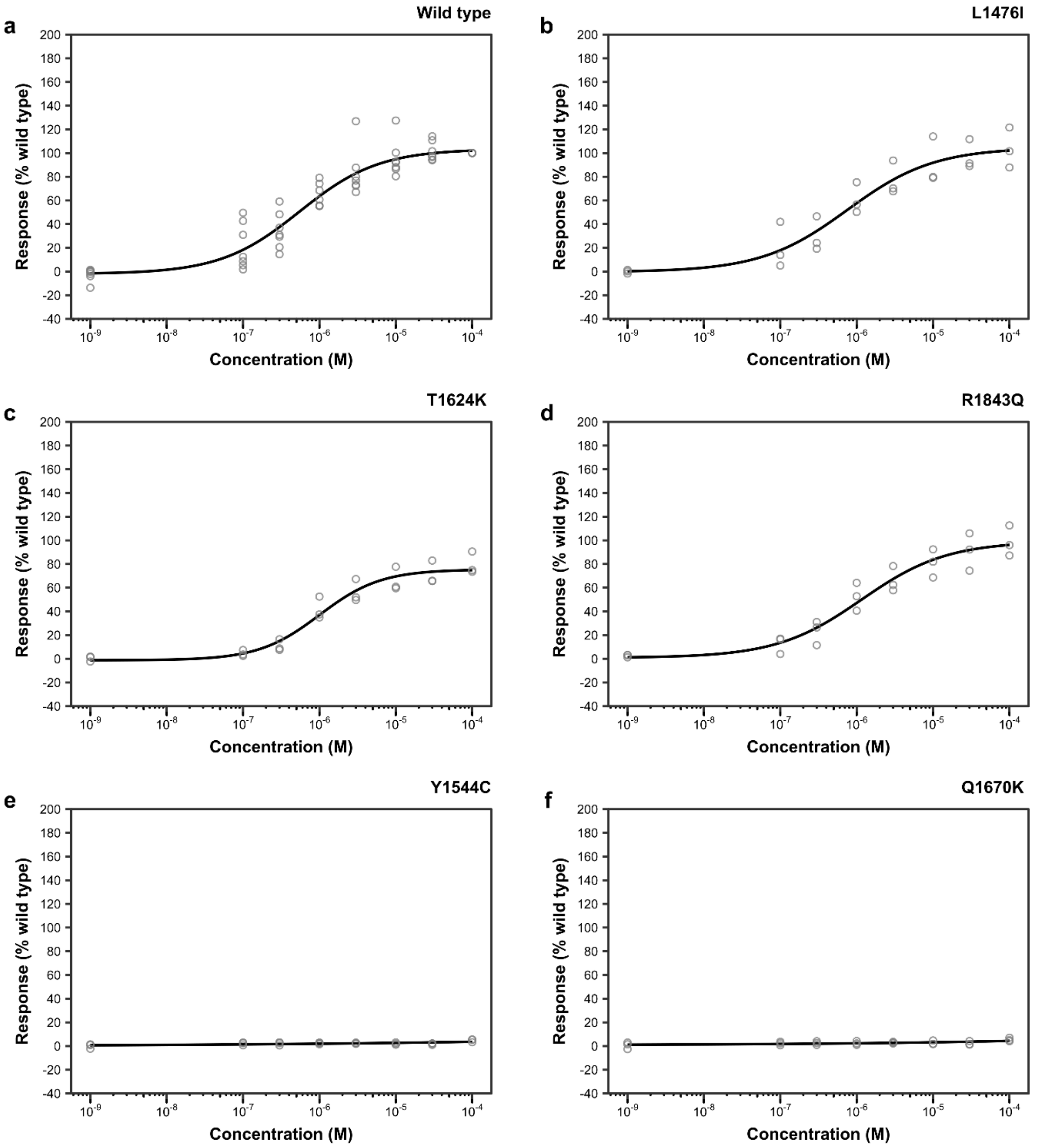

Figure S5. Functional profiling of *Sm*.TRPMPZQ variants.

Concentration-response relationships for the consensus *Sm*.TRPMPZQ sequence compared with six *Sm*.TRPMPZQ variants. **a)** Wild-type, **b)** L1476I, **c)** T1624K, **d)** R1843Q, **e)** Y1544C, **f)** Q1670K. Results represent the mean response relative to wildtype (Fig. 8a) from at least three independent transfections (points). The lines represent a fitted dose-response model using the four-parameter log-logistic function implemented in the drc package.

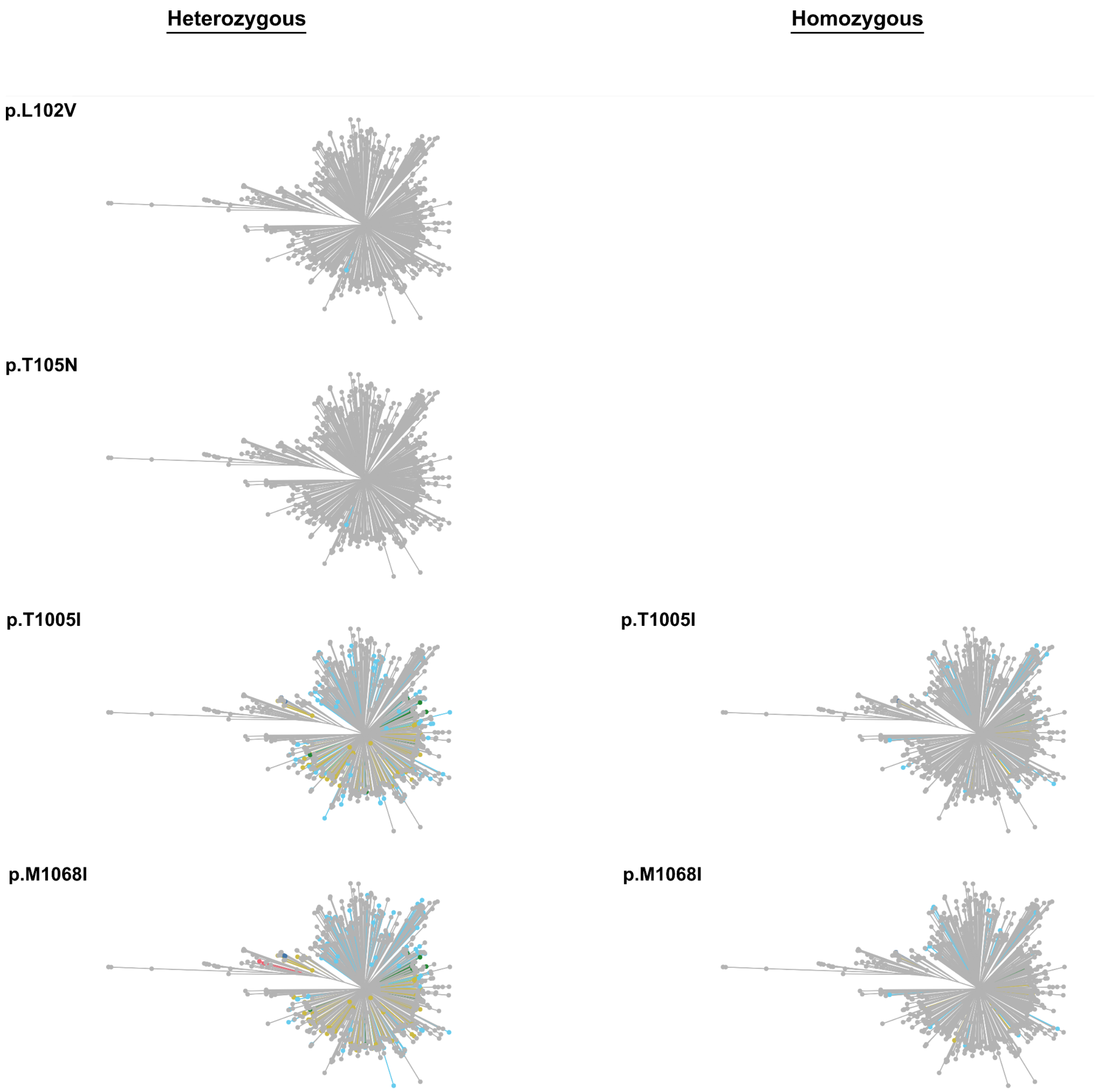

Figure S6. Distribution of individual mutations within *Sm.TRPM$_{PZQ}$* in analysed *Schistosoma* populations.

For each mutation (top left), non-grey lines and points represent accessions which contain the associated variants in either heterozygous or homozygous form. Colours correspond to sampling locations: Puerto Rico (PR; brown), Guadeloupe (GP; orange), Senegal (SN; dark purple), Cameroon (pink), Coastal Kenya (KE; teal), Lake Albert (dark blue), Eastern Uganda (red), Southern Uganda (light blue), Koome group islands (yellow), Northern Tanzania (dark green).

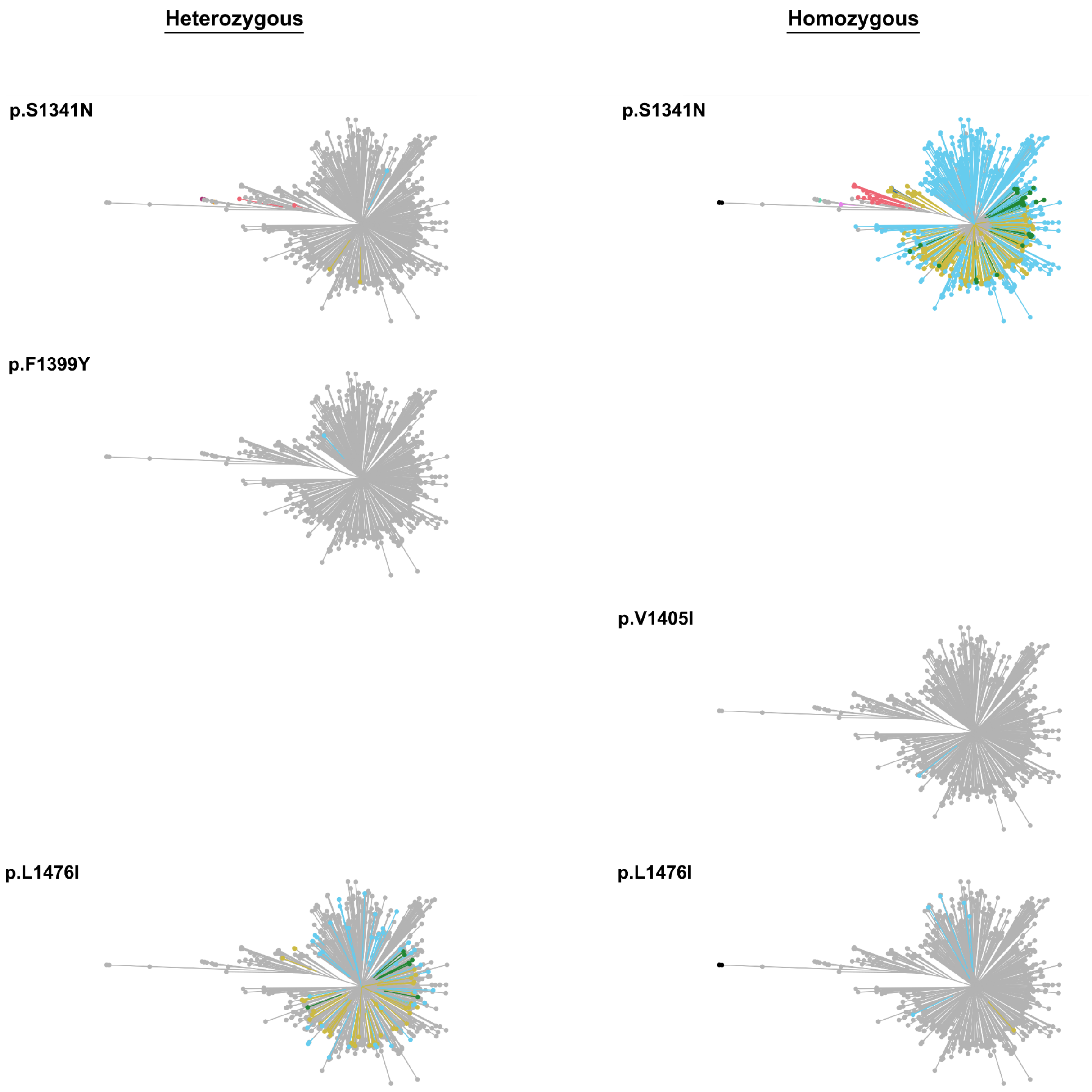

Figure S7. Distribution of individual mutations within *Sm.TRPM$_{PZQ}$* in analysed *Schistosoma* populations.
For each mutation (top left), non-grey lines and points represent accessions which contain the associated variants in either heterozygous or homozygous form. Colours correspond to sampling locations: Puerto Rico (PR; brown), Guadeloupe (GP; orange), Senegal (SN; dark purple), Cameroon (pink), Coastal Kenya (KE; teal), Lake Albert (dark blue), Eastern Uganda (red), Southern Uganda (light blue), Koome group islands (yellow), Northern Tanzania (dark green).

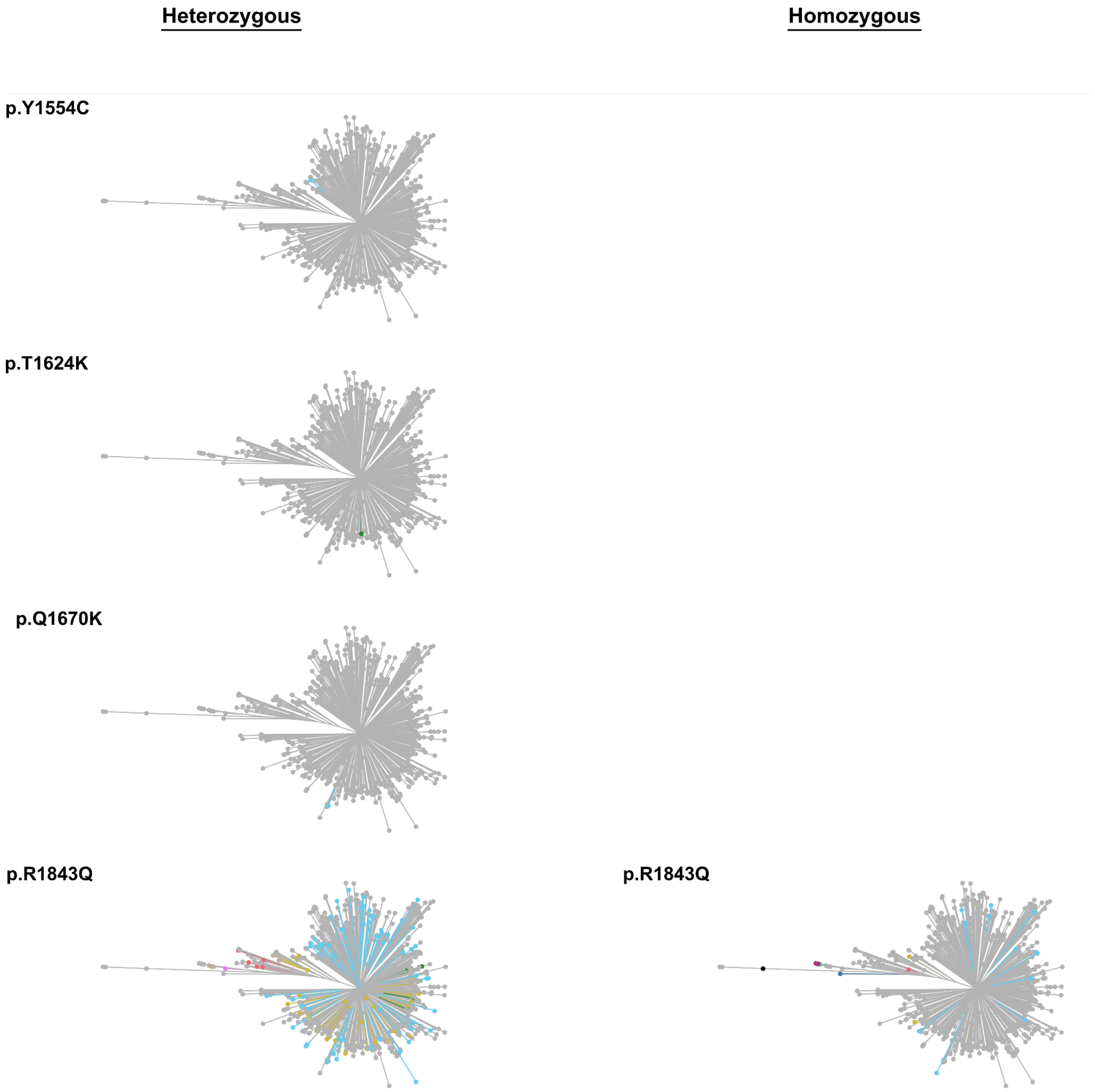

Figure S8. Distribution of individual mutations within *Sm.TRPM$_{PZQ}$* in analysed *Schistosoma* populations.

For each mutation (top left), non-grey lines and points represent accessions which contain the associated variants in either heterozygous or homozygous form. Colours correspond to sampling locations: Puerto Rico (PR; brown), Guadeloupe (GP; orange), Senegal (SN; dark purple), Cameroon (pink), Coastal Kenya (KE; teal), Lake Albert (dark blue), Eastern Uganda (red), Southern Uganda (light blue), Koome group islands (yellow), Northern Tanzania (dark green).

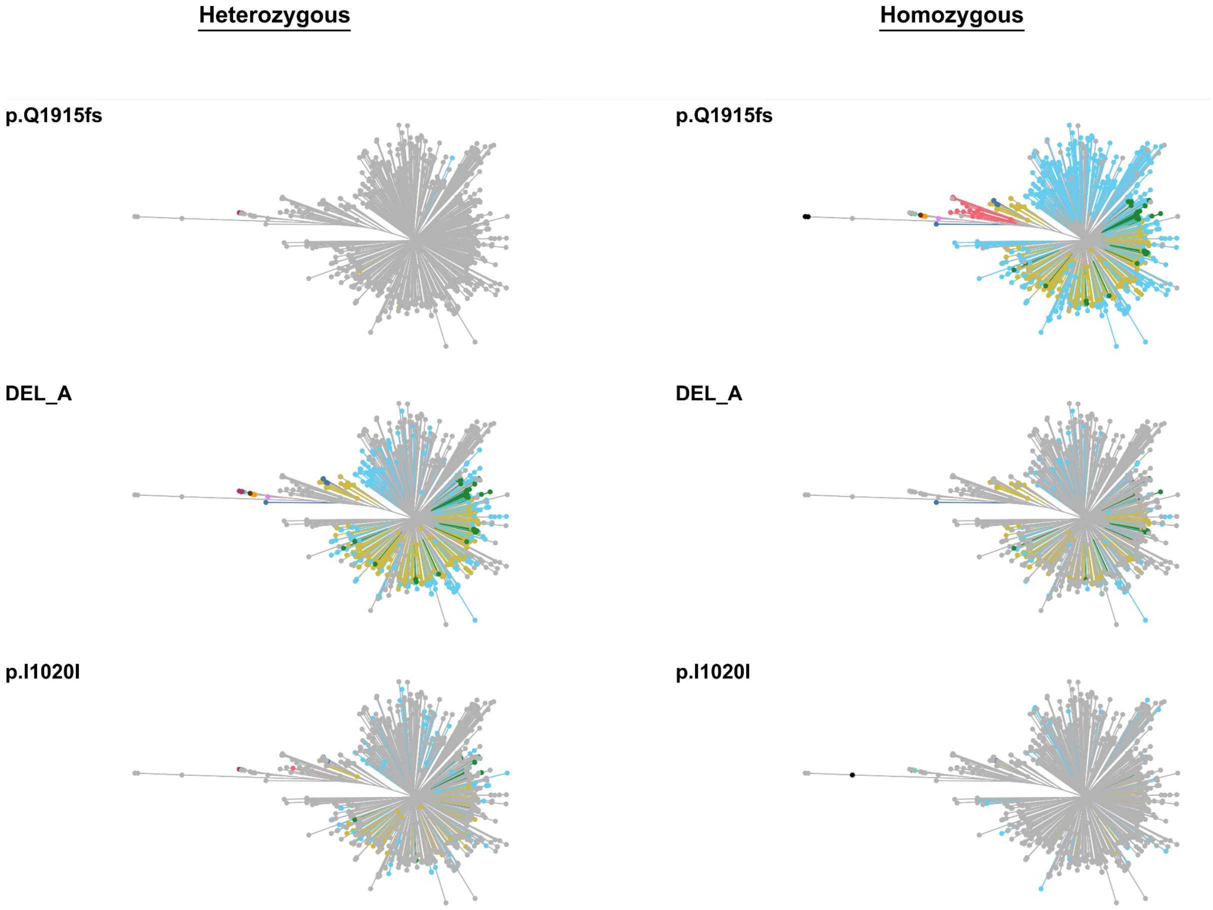

Figure S9. Distribution of individual mutations within or near *Sm.TRPM*$_{PZQ}$ in analysed *Schistosoma* populations.

For each mutation or structural variant (top left), non-grey lines and points represent accessions which contain the associated variants in either heterozygous or homozygous form. '150 kb deletion' refers to a series of 69.9-215.0 kb deletions located adjacent to Smp_345310 (~3.18-3.33 Mb on chromosome 3). Colours correspond to sampling locations: Puerto Rico (PR; brown), Guadeloupe (GP; orange), Senegal (SN; dark purple), Cameroon (pink), Coastal Kenya (KE; teal), Lake Albert (dark blue), Eastern Uganda (red), Southern Uganda (light blue), Koome group islands (yellow), Northern Tanzania (dark green).

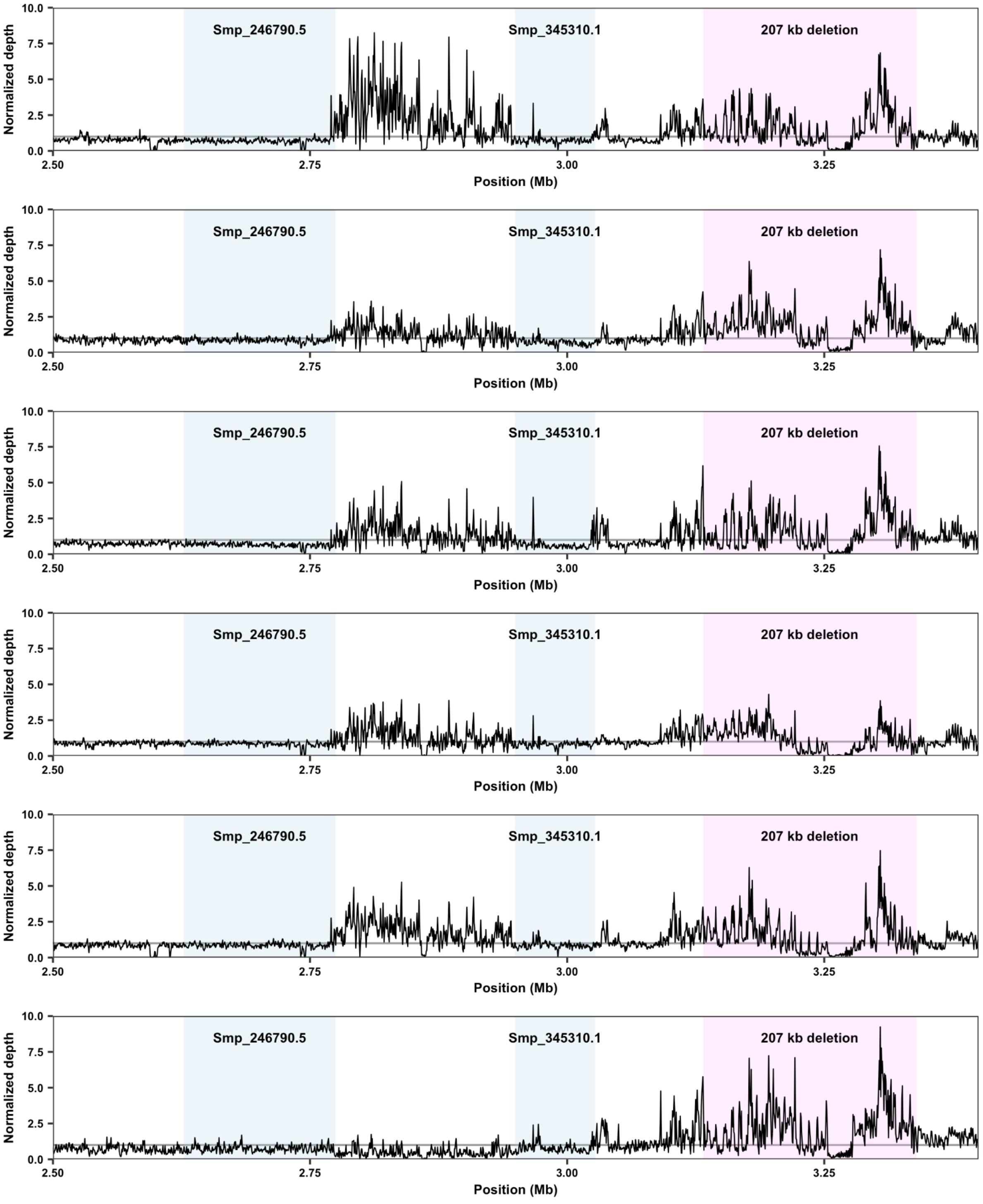

Figure S10. Depth of coverage of six accessions encompassing two genes implicated in reduced praziquantel susceptibility (Smp_246790.5, Smp_345310.1) and showing genotyped structural variants.

Light blue regions indicate the location of each gene, and pink regions indicate the range of the genotyped deletion. Black lines indicate the median depth of coverage in 500 bp windows divided by the average autosomal depth of coverage.

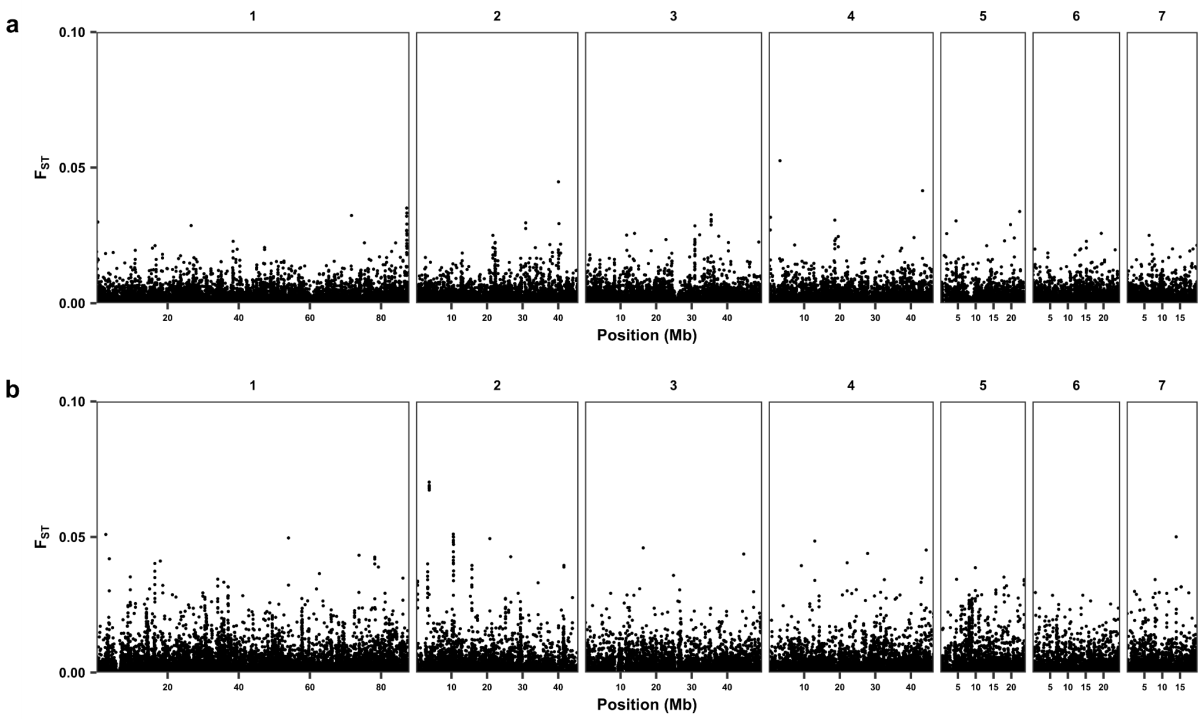

Figure S11. Genome-wide fixation index (F_{ST}) values between pre- and post-treatment populations.

Values were calculated between **a)** Southern Ugandan populations (*n* = 201 pre-treatment, *n* = 127 post-treatment) or **b)** Koome group island populations (*n* = 123 pre-treatment, *n* = 51 post-treatment) in 5 kb non-overlapping windows along each autosome. Points represent median F_{ST} values for each window.

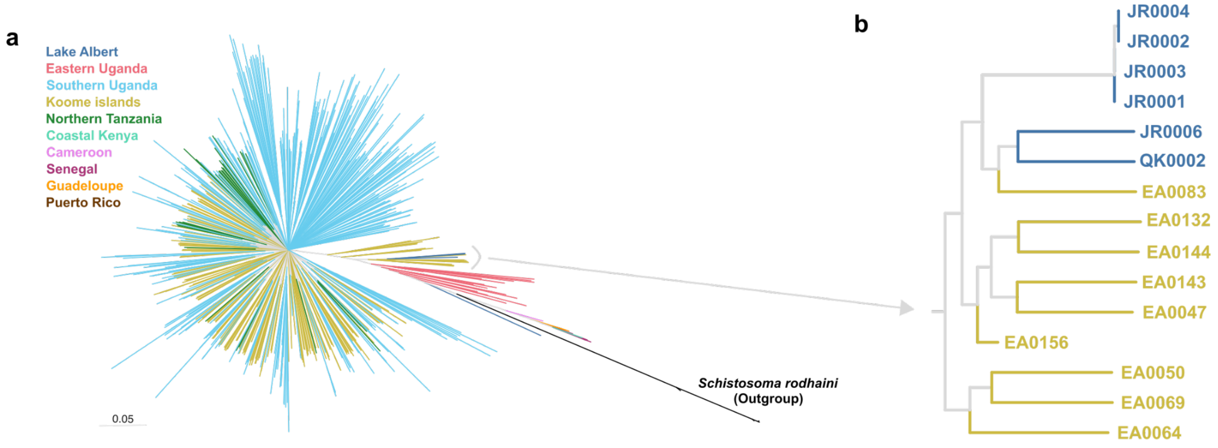

Figure S12. Phylogenetics of all accessions, with a focus on sample transmission within Uganda

a) Maximum-likelihood phylogenetic tree inferred using 188,923 autosomal single-nucleotide polymorphisms (SNPs) and all 574 accessions. Phylogenetic inference was conducted with IQ-TREE using a best-fit substitution model with ascertainment-bias correction. Branches are coloured based on the geographical region they were sampled from, and the tree is rooted on *S. rodhaini*. **b)** Highlighted clade containing samples potentially imported to the Koome group islands from Lake Albert.

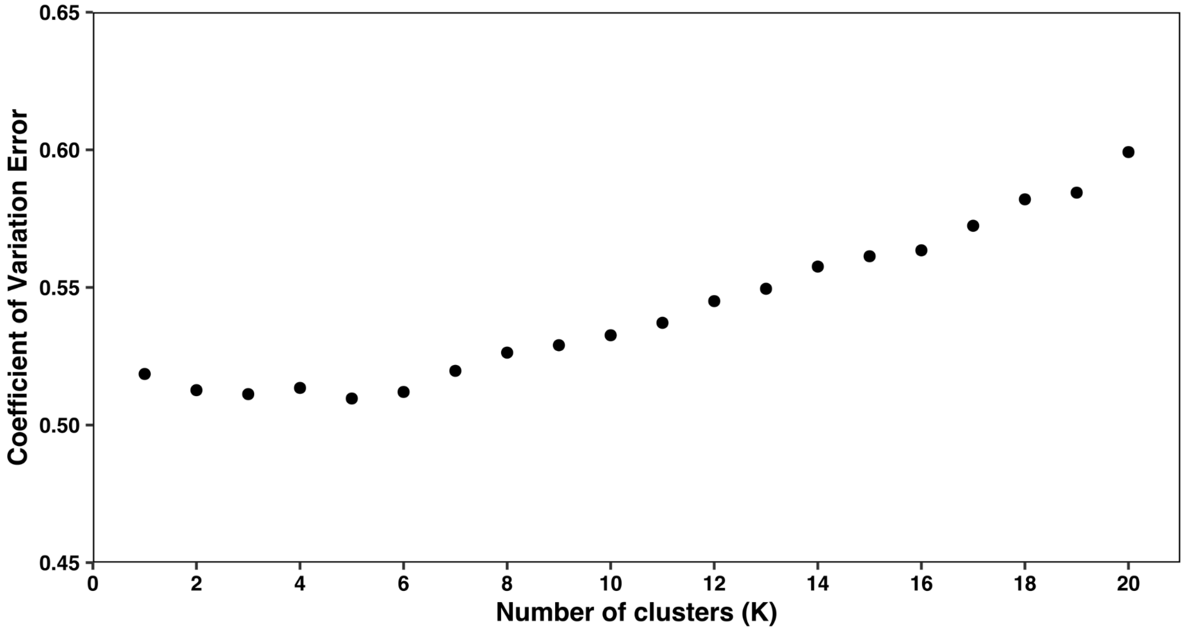

Figure S13. ADMIXTURE validation.
The coefficient of variation (CV) values generated by ADMIXTURE with K values ranging from 1 to 20, using 10-fold cross-validation and standard error estimation with 250 bootstraps, are shown. CV scores are shown for each K value.

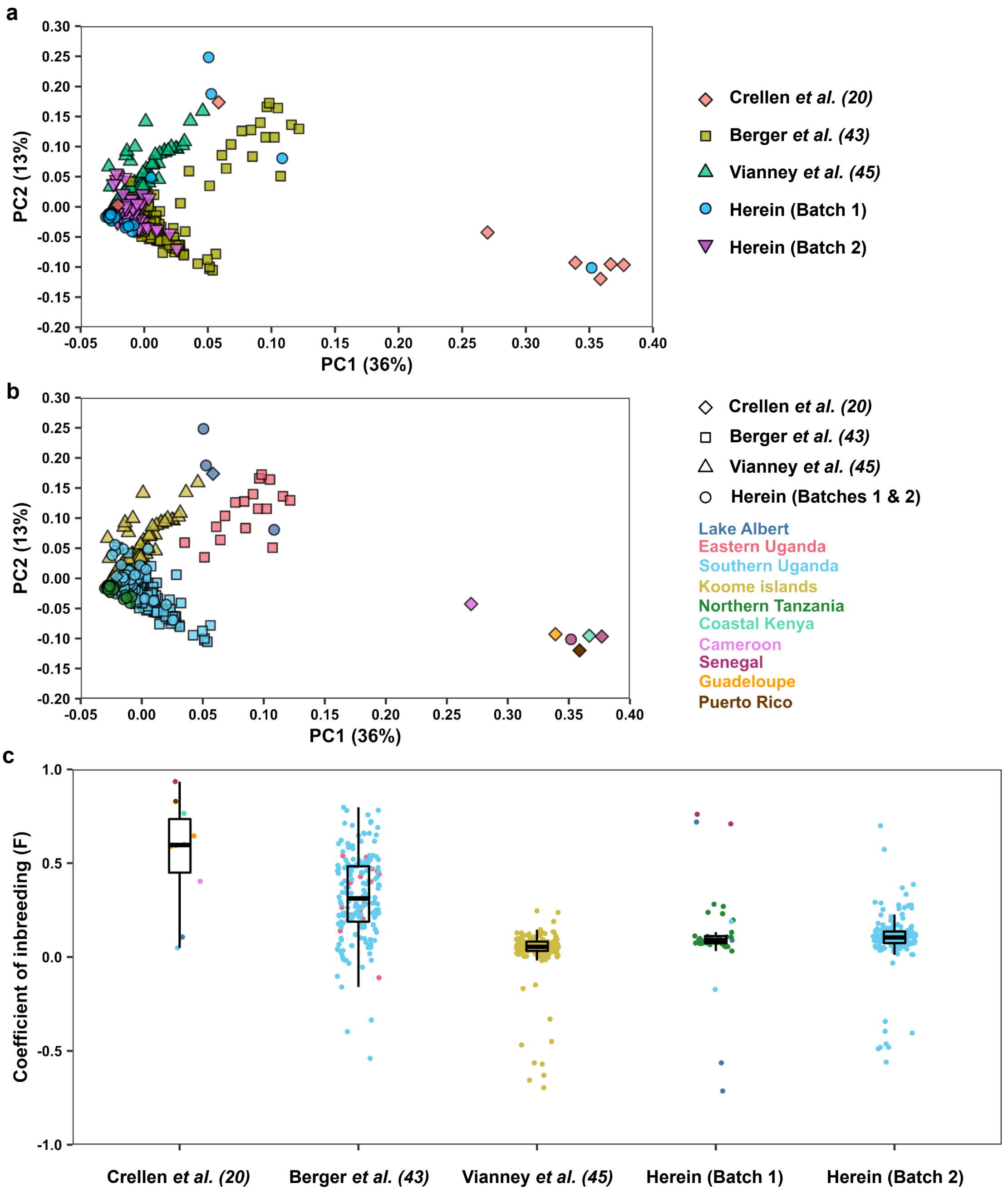

Figure S14. Population genetics and inbreeding.
Principal component analysis (PCA) of genetic differentiation between 505 unrelated *S. mansoni* accessions using 214,445 autosomal SNPs. Points are coloured and shaped by **a)** publication or **b)** by geographical origin and publication. **c)** Coefficient of inbreeding (F), points represent values for each accession, grouped by publication (x-axis) and coloured by sampling location as in **b)**. Batch 1: Samples with identifier prefix JR*, GN* or FS*, Batch 2: Samples with identifier prefix MK*.

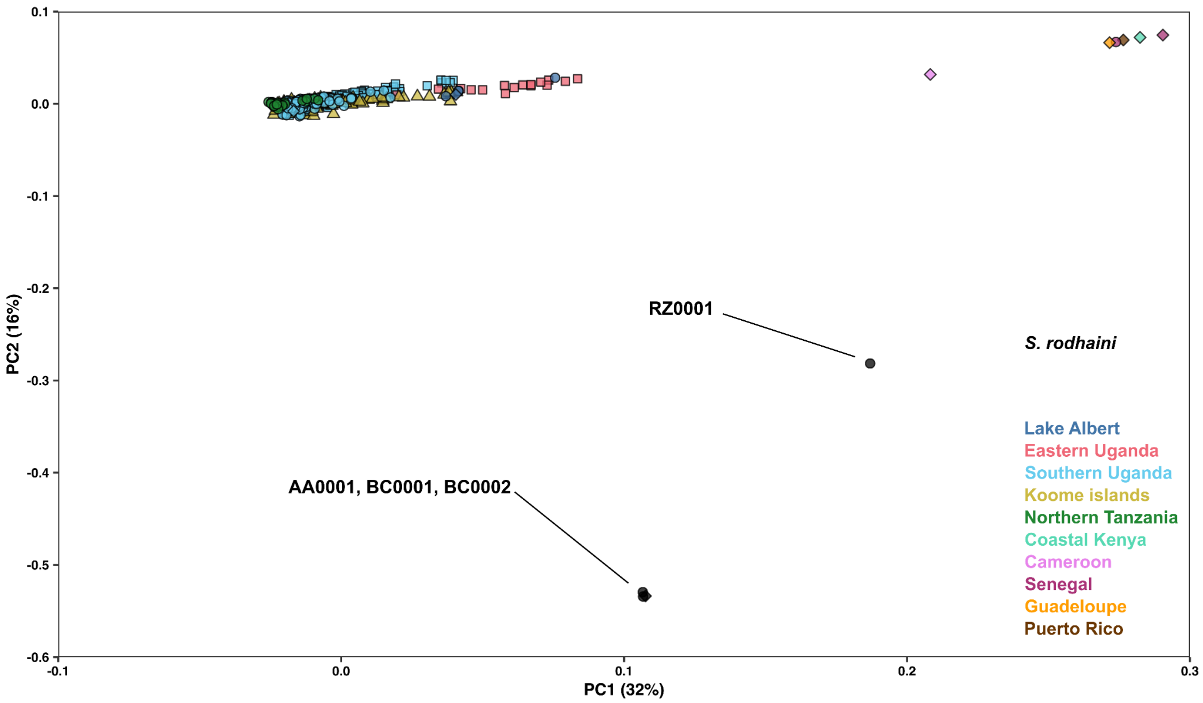

Figure S15. Comparison of outlier samples in the cohort.

Principal component analysis (PCA) of genetic differentiation between 505 unrelated *S. mansoni* (n = 542), *S. rodhaini* (n = 3; AA0001, BC0001, BC0002) or *S. mansoni-S. rodhaini* hybrid (n = 1; RZ0001) accessions using 201,213 autosomal SNPs. Points are coloured by geographical origin, point shapes represent the original publication from which each accession is derived: Crellen *et al. (20)* (diamond), Berger *et al. (43)* (square), Vianney *et al. (45)* (triangle), published herein (circle).

Supplementary Table Descriptions

Table S1: Accession metadata.

Table S2: Variant summary statistics. Per--sample summary statistics of post-quality control variant calls, as classified by BCFtools.

Table S3: Estimates of effective population size. Watterson's estimator (Θ) was calculated using Scikit-allel. Effective population size (Ne) was calculated using a per-generation mutation rate (μ) of 8.1e−9.

Table S4: Ancestry estimation. Per-sample ADMIXTURE results assuming five populations (K = 5). For accessions showing evidence of kinship, we selected one accession from each related cluster and excluded all others from analysis.

Table S5: Genome-wide integrated haplotype scores (iHS). Genome-wide integrated haplotype scores (iHS) were calculated independently using unrelated accessions from four populations. a) Southern Uganda (n = 287), b) Koome group islands (n = 159), c) Northern Tanzania (n = 30) and d) Eastern Uganda (n = 17). Columns include only windows with elevated iHS scores (|iHS|>2); windows within 50 kb of another window were grouped into continuous regions of selection.

Table S6: Genes within candidate regions of selection. Genes within continuous regions of elevated integrated haplotype scores (iHS) and their associated protein domains.

Table S7: Variants found within Smp_246790.5, for each accession. Genotyped variants within Smp_246790.5, listed per accession.

Table S8: Data and analysis from Ca2+ reporter assay profiling individual variants.

Table S9: Variants found within Smp_246790 (all isoforms), across all genotyped samples. Frequency of genotyped variants within Smp_246790.5.

Table S10: Variants found within Smp_246790.5. Frequency of genotyped variants within Smp_246790.5, subdivided by population and treatment stage.

Table S11: **Genotyped structural variants (deletions) across 389 samples.** Genotypes and genomic locations. SHQ (Smoove Het Quality), MSHQ for Mean SHQ uphold flank fold-change (DHFFC).

Table S12: Kinship across related accession comparisons tested. Estimation of relatedness between pairs of samples using NGSrelate. J9 (k0), J8 (k1), and J7 (k2) indicate the proportion of each genome where each pair of individuals shares 0, 1, or 2 alleles identical-by-descent, respectively. J1-J6 represent the remaining 6 Jacquard coefficients. rab is the pairwise relatedness, Fa is the inbreeding coefficient of accession 1, Fb is the inbreeding coefficient of accession 2, and theta is the coefficient of kinship. Further details can be found at: https://github.com/ANGSD/NgsRelate.

Table S13: Kinship across all accession comparisons. Estimation of relatedness between pairs of samples using NGSrelate. J9 (k0), J8 (k1), and J7 (k2) indicate the proportion of each genome where each pair of individuals shares 0, 1, or 2 alleles identical-by-descent, respectively. J1-J6 represent the

remaining 6 Jacquard coefficients. rab is the pairwise relatedness, Fa is the inbreeding coefficient of accession 1, Fb is the inbreeding coefficient of accession 2, and theta is the coefficient of kinship. Further details can be found at: https://github.com/ANGSD/NgsRelate.

Table S14: Read sequence metadata. Raw Illumina reads used for population genomic analyses, read locations, and associated metadata.
